# Can long-term historical data from electronic medical records improve surveillance for epidemics of acute respiratory infections? A systematic evaluation

**DOI:** 10.1371/journal.pone.0191324

**Published:** 2018-01-31

**Authors:** Hongzhang Zheng, William H. Woodall, Abigail L. Carlson, Sylvain DeLisle

**Affiliations:** 1 Department of Medicine, Veterans Affairs Maryland Health Care System, Baltimore, MD, United States of America; 2 School of Medicine, University of Maryland, Baltimore, MD, United States of America; 3 Department of Statistics, Virginia Polytechnic Institute and State University, Blacksburg, VA, United States of America; 4 Department of Infection Control and Hospital Epidemiology, Johns Hopkins Medical Institutions, Baltimore, MD, United States of America; University of Hong Kong, HONG KONG

## Abstract

**Background:**

As the deployment of electronic medical records (EMR) expands, so is the availability of long-term datasets that could serve to enhance public health surveillance. We hypothesized that EMR-based surveillance systems that incorporate seasonality and other long-term trends would discover outbreaks of acute respiratory infections (ARI) sooner than systems that only consider the recent past.

**Methods:**

We simulated surveillance systems aimed at discovering modeled influenza outbreaks injected into backgrounds of patients with ARI. Backgrounds of daily case counts were either synthesized or obtained by applying one of three previously validated ARI case-detection algorithms to authentic EMR entries. From the time of outbreak injection, detection statistics were applied daily on paired background+injection and background-only time series. The relationship between the detection delay (the time from injection to the first alarm uniquely found in the background+injection data) and the false-alarm rate (FAR) was determined by systematically varying the statistical alarm threshold. We compared this relationship for outbreak detection methods that utilized either 7 days (early aberrancy reporting system (EARS)) or 2–4 years of past data (seasonal autoregressive integrated moving average (SARIMA) time series modeling).

**Results:**

In otherwise identical surveillance systems, SARIMA detected epidemics sooner than EARS at any FAR below 10%. The algorithms used to detect single ARI cases impacted both the feasibility and marginal benefits of SARIMA modeling. Under plausible real-world conditions, SARIMA could reduce detection delay by 5–16 days. It also was more sensitive at detecting the summer wave of the 2009 influenza pandemic.

**Conclusion:**

Time series modeling of long-term historical EMR data can reduce the time it takes to discover epidemics of ARI. Realistic surveillance simulations may prove invaluable to optimize system design and tuning.

## Introduction

Outbreaks due to novel strains of influenza [1–3] or coronavirus [4, 5] illustrate why we must remain vigilant toward epidemics of acute respiratory infections (ARI). ARI epidemics need be recognized as soon as possible if prevention and mitigation measures are to be effective [6, 7].

The electronic medical record (EMR) includes numerous entries (e.g. clinical notes, vital signs, diagnostic codes, test results) that could combine to facilitate the discovery of individual cases [8, 9] or outbreaks of ARI [10]. In integrated health delivery systems, EMR data originate across the continuum of care, from ambulatory visits to intensive care units. A monitoring system rooted in such a comprehensive EMR implementation could provide early insight into outbreak severity and support the flow of information necessary to manage specific patients as well as the overall epidemic [11–13].

With the EMR fast becoming commonplace [14], long-term EMR-derived datasets will soon be routinely available. In this context, we asked if statistical methods that consider long-term EMR data patterns could benefit ARI epidemic detection. Because most ARI result from viral diseases that exhibit marked seasonality, we hypothesized that fitting multi-year time-series of daily ARI case counts with seasonal autoregressive integrated moving average (SARIMA) models [15, 16] could accelerate epidemic detection, compared to methods that base their forecasting on the recent past only [17]. To begin to test this hypothesis, we used software to recreate surveillance systems operating prospectively on synthetic or authentic historical datasets. Our results suggest that, under realistic surveillance conditions, SARIMA could help shorten outbreak detection delay.

## Methods

### Ethics statement

The Institutional Review Boards of the Veterans Administration (VA) Maryland Health Care System and the University of Maryland approved this study. The study was granted a waiver of consent, as risks were limited to information confidentiality, because the research-related risks were minimal and did not adversely affect the rights and welfare of the participants, and because the work would not have otherwise been feasible, given the large number of participants. All EMR information was de-identified prior to simulations and analyses, which used only daily case counts.

### Description of procedures

#### Synthetic backgrounds and outbreaks

Simulated background time series of daily case counts were obtained from the Centers for Disease Control and Prevention website (http://www.bt.cdc.gov//surveillance/ears/.datasets.asp, accessed February 20^th^, 2010). We chose the s17 and the s33 time series, which mimic typical surveillance data for ARI such as pneumonia, influenza or influenza-like illness, with lower counts on weekend and holidays, long-term increase in counts and seasonality [18]. The synthetic outbreaks “08” (Log Normal) or the “04” (Inverted Log Normal) served as the epidemic signal to be injected. Characteristics of the synthetic backgrounds are summarized in Table 1. The datasets are provided as supplementary material (S1 File).

**Table 1:** Characteristics of the background time series.

Origin	Weekdays		Weekends	
	Mean	Standard Deviation	Mean	Standard Deviation
s17	182.95	128.12	184.92	126.56
s33	34.09	8.53	33.75	8.61
CDA1	29.41	13.36	6.28	3.45
CDA2	6.99	5.38	2.05	2.04
CDA3	0.70	0.88	0.68	0.98

#### Authentic backgrounds

**Fig 1:**
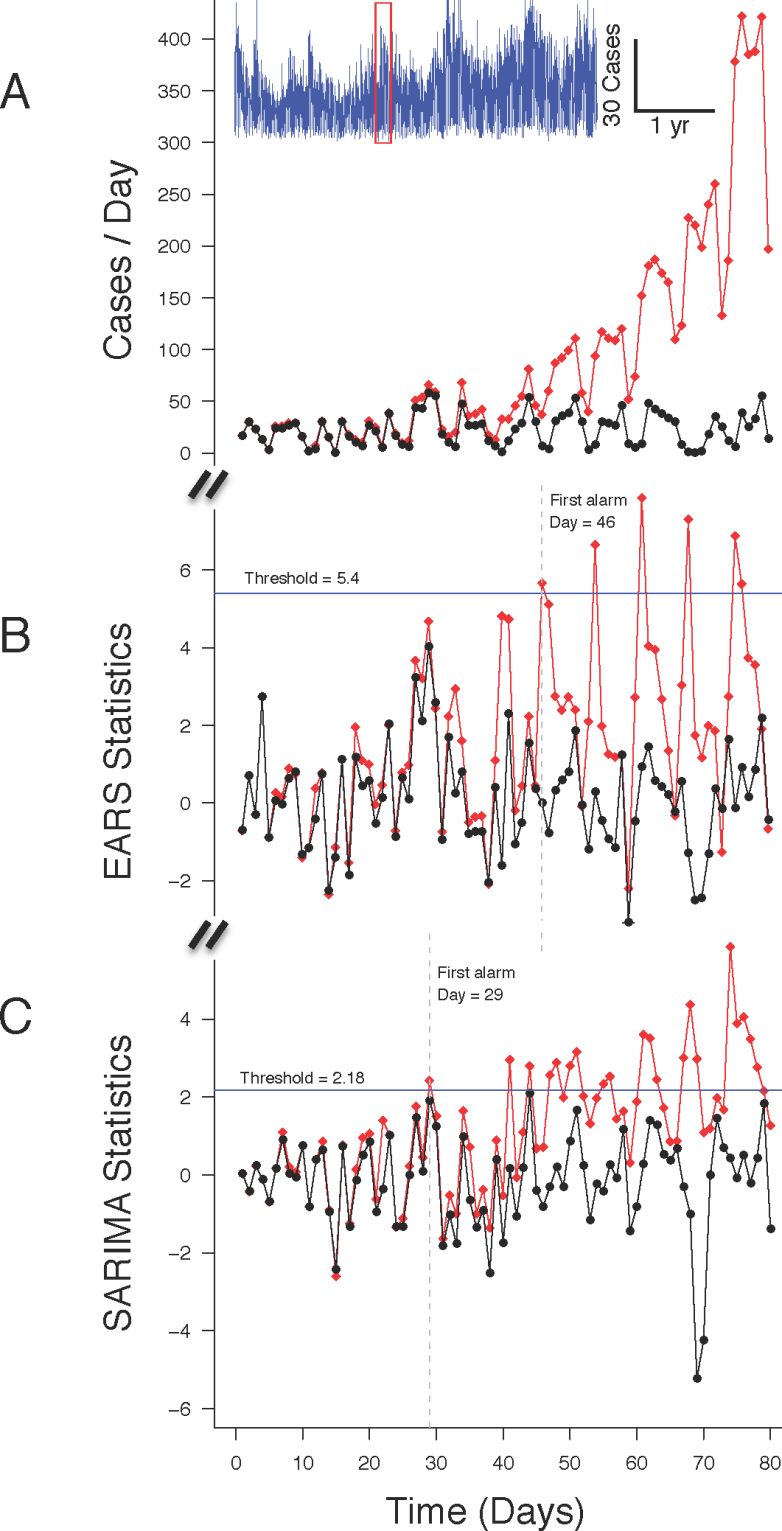
Comparing surveillance systems performance. Y-axis shows daily case counts (panel A) and corresponding EARS (panel B) and SARIMA statistics (panel C) as a function of time (x-axis) for an 80-day period extracted from a multiyear time series of authentic ARI cases (red rectangle region from the blue tracing insert in panel A). Black circles plot values due to background ARI cases only; red diamonds plot values due to background + injected epidemic cases. Note that at equivalent thresholds, corresponding to a 1% FAR (horizontal blue lines in panels B and C), the first true positive alarm (vertical dashed lines in panels B and C) occurs earlier with SARIMA (day 29, panel C) than with EARS (day 46, panel B).

EMR entries were extracted from Veterans Integrated Service Technology Architecture hierarchical databases (MDE v.6.1.0.0, Strategic Reporting Systems, Inc. Peabody MA) and transferred to relational databases (SQL Server 2008, Microsoft Corp, Redmond CA). We used case detection algorithms (CDA) previously developed against a manual record review seeking reference ARI cases, defined as: [Positive influenza culture/antigen OR Any two of the following, of no more than 7 days duration: cough; fever or chills or night sweats; pleuritic chest pain; myalgia; sore throat; headache] AND Illness not attributable to a non-infectious etiology [8]. We chose the following ARI CDAs: a) CDA1 (sensitivity 63%, positive predictive value (PPV) 13%) retrieved outpatient visits where provider assigned a diagnostic code (International Classification of Diseases, 9^th^ version) included in an ARI-related set used by a surveillance system of national scope [19]; b) CDA2 (sensitivity 69%, PPV 54%) retrieved outpatient visits assigned a diagnostic code that belonged to a grouping modified for increased performance at the VA [8]), and where automated text analysis of the visit-related clinical note(s) identified at least two symptoms from the above ARI case definition [8, 20]. CDA2 was chosen because its use resulted in the highest epidemic detection performance amongst eight (8) alternative ARI case-detection methods [10], illustrating the gains in system performance that could be achieved by combining different EMR data fields compared to using diagnostic codes alone; c) CDA3 retrieved CDA2 patients subset with a core body temperature of more than 37.8°C measured on the day of their index visit (sensitivity 71%, PPV 68%). Our rationale for choosing CDA3 was that while its high performance could translate into timely epidemic detection, low daily case counts could represent a challenging substrate for time series modeling. To generate CDA-specific time series of daily case counts, each ARI CDA was applied to a relational database containing EMR documentation of outpatient clinical encounters [8] over a 7-year period, from January 1999 to December 2005. These time series formed the basis for the authentic “backgrounds” used for the whole-system simulations (see Fig 1A (insert), Table 1, and S1 File). EMR data included in CDA 1–3 have been extracted daily at the VAMHCS. Thus, any of the CDA tested could be incorporated in a prospective surveillance system.

#### Modeled influenza epidemic

We developed an epidemic model of influenza to supply a plausible outbreak signal with which we could compare alternative surveillance system constructs [10]. The model includes coupled series of differential equations [21] adjusted to describe an epidemic of severe, 1918-like influenza, propagating through a susceptible population with the size, age structure, and age-specific birth and death rates described for Baltimore, MD [10]. The proportion of community cases presenting to the VA Maryland Health Care System was adjusted to reflect the age, gender, and population estimates of Baltimore veterans, over half of whom are older than 60 years old and more than 90% are male [22]. The model, its settings and documentation are provided as S2 File). Prior to injection, the modeled epidemic case counts were first discounted by the known sensitivity of the ARI CDA used to generate the background cases [8]. Fig 1A shows an example of the modeled influenza outbreak injected into an authentic background.

#### Simulations of surveillance systems

We wrote software to simulate prospective surveillance systems operating on the authentic historical background datasets described above (R v. 2.10.1, http://www.r-project.org). The software began a surveillance cycle on the day when the modeled epidemic signal was injected into a background time series. Starting on the injection day, and then daily for a total of 80 days, a statistical outbreak detection method (see below) was applied in parallel to the background+epidemic (Combined) time series, and to the “Background-only” time series. The value of the statistic for each day was stored for subsequent analysis. The 80-day surveillance cycle was repeated, each time shifting the outbreak injection to a different week of the two, one-year study periods (from August 1^st^ 2002 to July 31^st^ 2003, and from August 1^st^ 2003 to July 31^st^ 2004). In a final phase, the software applied a family of threshold values beyond which the computed daily statistics would constitute an “alarm”. On a given day and for a given threshold, an alarm that originated in the Background-only time series was considered a “false alarm”. An alarm present in the Combined dataset but absent from the Background-only dataset was considered a “true alarm” (Fig 1B and 1C). For a given alarm threshold, the software computed two benchmarks: 1) the “Detection Delay”, the time from outbreak injection to the first true alarm, averaged for the 52 weekly surveillance cycles for a given one-year study period; 2) the daily false alarm rate (FAR), defined as the number of unique false alarms during the evaluation year, divided by 365 days. Corresponding pairs of Detection Delay and FAR were computed over broad ranges of alarm thresholds and used to plot CDA-specific activity monitoring operating characteristic (AMOC) curves [23].

#### Outbreak detection statistics

We compared two statistical approaches to detect the addition of the modeled epidemic signal to the backgrounds: 1) the early aberration reporting system (EARS) W2c [24], which made predictions using data from the past 7 days of the relevant time series; and 2) seasonal autoregressive integrated moving average (SARIMA) models [25], which used 2–4 years of past data.

*EARS*. Daily case counts were first separated into two time series, one for weekdays and another for weekends/federal holidays. For a given day, the W2c statistic is expressed as

$$W2c(t)=\frac{Y(t)-\overset{-}{Y}(t)}{S(t)},$$

where *t* is the time series index, *Y(t)* is the observed case count on that index day, $\bar{Y}(t)$ and *S(t)* are a 7-day moving sample mean and standard deviation calculated with a 2-day lag from the index day. The value of *S(t)* was replaced by 1 if *S*(*t*) < 1.

*SARIMA*. We used the Box-Jenkins method [15, 26] to develop separate SARIMA models (SAS v. 9.1, SAS Institute Inc. Cary NC) for the weekdays and weekends background time series corresponding to the s17 and s33 datasets (January 1^st^ 1994 to December 31^st^ 1997) or retrieved via each of the three ARI CDAs (from January 2, 1999 to August 13, 2002). Models were of the form “SARIMA*(p*,*d*,*q)(P*,*D*,*Q)s*”, which abbreviates the following equation:

$$\Phi_{P}^{*}(B^{s})\Phi_{p}(B){\left(1-B\right)}^{d}{\left(1-B^{s}\right)}^{D}y_{t}=\Theta_{Q}^{*}(B^{s})\Theta_{q}(B)\varepsilon_{t},$$

where *B* is the backshift operator; *y_t_* is the observation at time *t*; *ε_t_* is a sequence of independently distributed random shocks assumed to be normally distributed with a mean of zero and constant variance *σ*^2^; *Φ_p_*(*B*) = (1 − *ϕ*_1_*B* − *ϕ*_2_*B*^2^ − ⋯ − *ϕ_p_B^p^*) is an autoregressive polynomial of order *p* for non-seasonal component; *d* is the order of the differencing of the non-seasonal component; *Θ_q_*(*B*) = (1 − *θ*_1_*B* − *θ*_2_*B*^2^ − ⋯ − *θ_q_B^q^*) is a moving average polynomial of order *q* for the non-seasonal component; $\Phi_{P}^{*}(B)=(1-\phi_{1}^{*}B^{s}-{\phi_{2}^{*}B}^{2s}-\cdots -\phi_{p}^{*}B^{Ps})$ is an autoregressive polynomial of order *P* for the seasonal component; *D* is the order of the seasonal differencing; and $\Theta_{Q}^{*}(B)=(1-\theta_{1}^{*}B^{s}-{\theta_{2}^{*}B}^{2s}-\cdots -{\theta_{Q}^{*}B}^{Qs})$ is a moving average polynomial of order *Q* for the seasonal component. The cyclic periods were taken as 364 days [27], 260 days and 104 days for years, weekdays and weekends, respectively. Before model-fitting, times series were modified as follows: 1) occasional high counts (n = 2–4 per year) and low counts for federal holidays were replaced by the average of the past three daily counts from the relevant time series; 2) a square root transformation was applied to make the data series stationary in both mean and variance, and thus satisfy model assumptions [16, 26].

Putative models were explored interactively to search for the most appropriate SARIMA form for each time series. The models coefficients were then estimated and their statistical significance evaluated. Some coefficients of the autocorrelation function (ACF) and the partial autocorrelation function (PACF) at lags of 260 and multiples of 260 were statistically significant, confirming that seasonal model components should be included. Overall model adequacy was assessed by comparing model-generated counts to the actual counts used for model building. SARIMA models were finally retained for surveillance implementation when they best fulfilled the following criteria: 1) minimum Akaike's Information Criterion value; 2) minimum mean absolute deviation of errors; 3) minimum root mean square root error; 4) normality of residuals; 5) parsimony. Each of the sixteen SARIMA models was used daily to forecast the next day’s case count in their respective times series during the study periods. With each passing day, the models updated themselves to incorporate the latest information into the forecasting procedure.

The final SARIMA models for weekday ARI counts were of the form SARIMA (1,0,1)(0,1,1)_260_ for CDA1 and CDA2, but were of a different form for the synthetic backgrounds i.e. SARIMA (0,1,1)(1,0,1)_260_ and SARIMA (1,0,1) (1,0,1)_260_ for the s17 and the s33 time series, respectively. Models for the weekend ARI counts were all of the form SARIMA (1,0,1)(0,1,1)_104_. Case counts retrieved by CDA3 were low, and did not allow SARIMA modeling.

## Results

### Synthetic context

**Fig 2:**
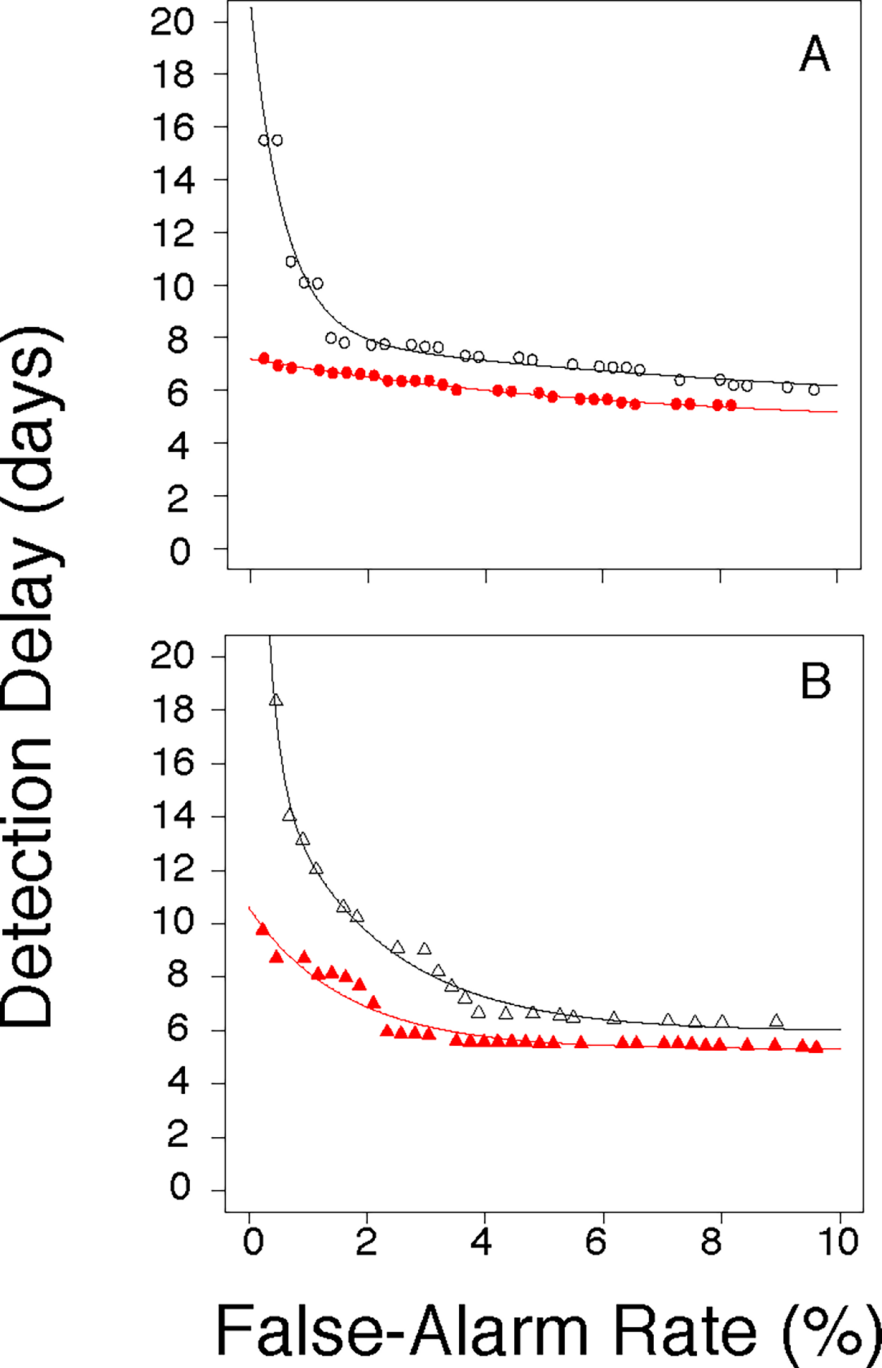
Performance comparisons: Synthetic contexts. Activity monitoring operating characteristic (AMOC) curves showing average detection delay (y-axis) as a function of daily false alarm rate (x-axis) for discovering a synthetic outbreak signal introduced into the s17 (panel A) or s33 (panel B) synthetic background time series of daily case counts. Aberrancy detection was performed using either EARS (open black symbols) or SARIMA (closed red symbols).

In an initial set of simulations, we utilized either the EARS or the SARIMA outbreak detection methods on synthetic time series that exhibited seasonal increases in case counts typical of ARI. As expected, lowering the statistical alarm threshold decreased the time it took to discover the injected outbreak but increased the false-alarm rate (FAR). As the AMOC curves shown in Fig 2 illustrate, the relationship between the detection delay and the FAR was not linear, with a change around a low FAR producing a greater change in outbreak detection delay than a similar change around a high FAR.

The SARIMA method discovered the injected outbreaks faster than EARS did, at any FAR below 10% (compare EARS (open black symbols) to SARIMA (closed red symbols) for the s17 (Fig 2A) or the s33 backgrounds (Fig 2B)). The timeliness advantage of SARIMA over EARS became increasingly pronounced at FARs below 2%, where real-world surveillance systems would be expected to operate.

### Semi-synthetic context

To determine if the performance gains observed with SARIMA in all-synthetic contexts would translate into meaningful real-world benefits, we modified the simulations in two ways: 1) we ran previously validated ARI CDAs on a repository of our own EMR data to generate authentic historical time series of ARI cases [8]; 2) we modeled an epidemic of severe influenza centered in Baltimore to create an ARI outbreak that could plausibly be experienced by our health system. To gain insight into year-to-year variation in system performance, we performed surveillance simulations over two different one-year time periods: a) 8/2002-7/2003, a year with an average ARI incidence; and b) 8/2003-7/2004, a year with a high seasonal incidence of ARIs.

**Fig 3:**
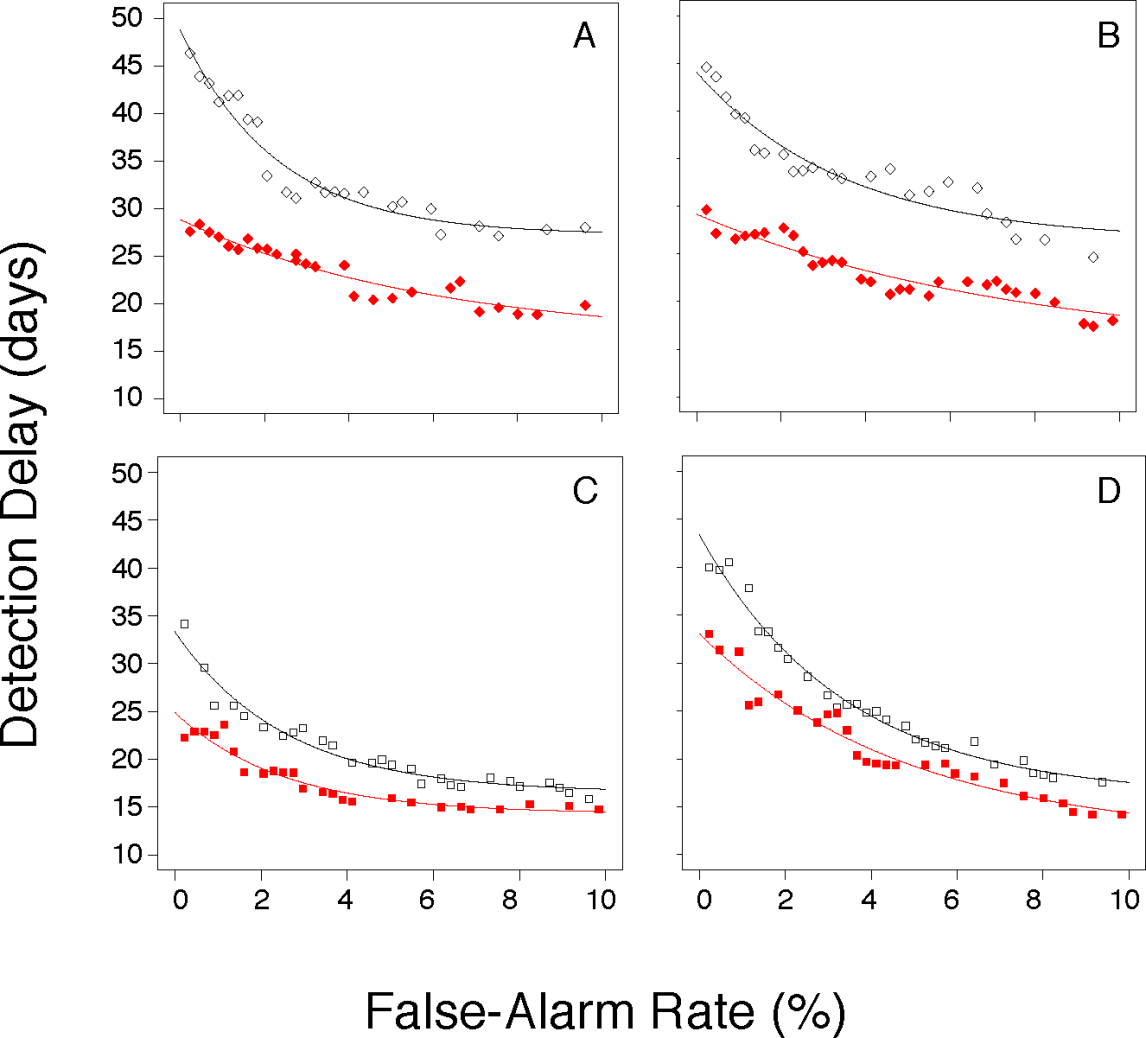
Performance comparisons: More realistic, semi-synthetic context. AMOC curves describing the performance of surveillance systems at detecting a modeled influenza epidemic injected into authentic backgrounds obtained by implementing CDA1 in 2002–2003 (panel A) or in 2003–2004 (panel B), and CDA2 in 2002–2003 (panel C) or in 2003–2004 (panel D). Outbreak detection methods were either EARS (open black symbols) or SARIMA (closed red symbols).

In keeping with prior observations [10], EARS-based surveillance exhibited a better performance when using CDA2 for ARI case detection than when using CDA1, with consistently shorter outbreak detection delay at any FAR for both study periods (in Fig 3, compare CDA1 (open black diamonds) to CDA2 (open black squares) across panels A and C (2002–03) and across panels B and D (2003–04).

SARIMA outbreak detection consistently outperformed EARS for both ARI CDAs (for CDA1, compare open black to closed red diamonds in Fig 3A (2002–03) and Fig 3B (2003–04); for CDA2, compare open black to closed red squares in Fig 3C (2002–03) and Fig 3D (2003–04)). Gains in timeliness due to SARIMA were highest at low FAR and were larger for CDA1 than for CDA2 (in Fig 3, compare the distance between the black and red tracings in panels A and B to that in panels C and D). Examination of the AMOC curves in Fig 3 suggests that at the commonly used daily FAR of 1%, SARIMA could reduce detection delay by ~14–16 days for CDA1, and by ~5 days for CDA2.

### Real-world context: The 2009 influenza pandemic

To gain further insight into the practical usefulness of incorporating long-term historical data for ARI surveillance, we extended the CDA2-based surveillance system forward in time to include the summer of 2009, when the first wave of a pandemic of a novel H1N1 influenza strain (pH1N1) reached the US [2]. We avoided the time period immediately following the public announcement of the pandemic, as this period contained many visits from worried individuals but only sporadic laboratory-confirmed pH1N1 cases. Our comparison period thus began on May 24 and ended August 22, before the very large second wave of the epidemic reached Baltimore. During this period, there were peaks in laboratory-based surveillance for pH1N1 in Baltimore, MD, but no corresponding local peak of ARI visits observed in CDC's BioSense syndromic surveillance system.

**Fig 4:**
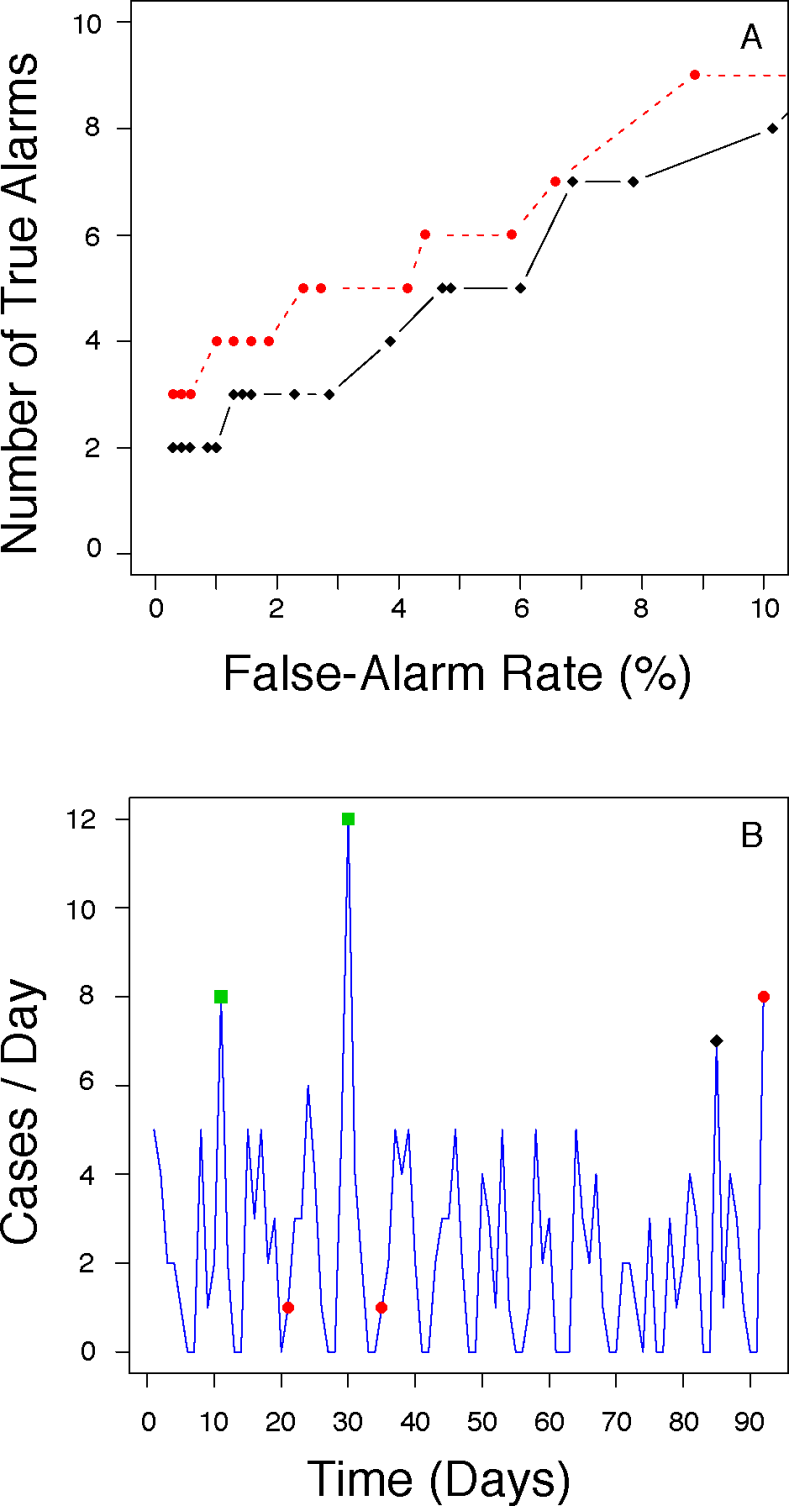
Performance comparisons: pH1N1 pandemic. Panel A: Number of true-positive alarms (y-axis) as a function of daily FAR (x-axis) for the full summer of 2009 comparison period. Note that SARIMA (red circles) issued more true alarms than EARS (black diamonds) at any FAR. Panel B: Daily counts of authentic ARI cases (y-axis) during the summer of 2009 comparison period (x-axis). True alarms at a 3% FAR were issued by SARIMA alone on the days highlighted by red circles, EARS alone (black diamond) or by both SARIMA and EARS (green squares). Note that the first alarm of the time period is recognized by both statistical approaches (see text).

During the summer 2009 comparison period, SARIMA identified more true-positive alarms than EARS at any FAR below 10% (Fig 4A). The example shown in Fig 4B highlights additional true positive alarms uncovered by SARIMA that were missed by EARS (red dots). Within this FAR range, both EARS and SARIMA identified the first true positive alarm, a large peak of ARI cases occurring 10 days into the comparison period. Thus, for this particular outbreak signal, the more sensitive SARIMA method offered no timeliness advantage over EARS.

## Discussion

In this work, we found that modeling long-term trends and seasonality in disease incidence with SARIMA can improve the performance of EMR-based influenza surveillance compared to EARS, a method that considers the recent past only. The algorithms used to detect single ARI cases impacted both the feasibility of SARIMA modeling and the magnitude of the SARIMA-related gains in detection timeliness. Under plausible conditions for our local health system, SARIMA could speed up epidemic detection by several days, a meaningful improvement considering how rapidly influenza can spread through a community [28].

If the need for a software framework to assess surveillance systems has previously been recognized [29], we could find no published precedent for a modular test bed where both case- and outbreak-detection methods can be interchanged. Because adjustments in statistical alarm thresholds are method-specific and their effect on FAR unpredictable, we used simulations to determine the relationship between FAR and detection delay empirically. A graphical representation of this relationship intuitively informed a performance trade-off of key concern to public health practitioners.

Many statistical aberrancy detection methods have been described for epidemic surveillance [17, 30–34], including time series modeling [31, 35, 36]. Some of these methods have been applied to the same data sets and formally compared [18, 37–40]. Of the two studies that addressed the value of historical data, a generalized linear model (GLM) that took advantage of 3 years of past data often outperformed EARS [39], whereas a seasonally adjusted cumulative sum (CUSUM) method did not [18]. Even though a number of explanations could help reconcile these results, we note that as the size of an epidemic signal increases relative to the background noise, outbreak recognition becomes easier and the performance of alternative detection methods becomes more and more difficult to distinguish [39, 41]. This rationale could explain why the GLM approach implemented by Jackson et al. did not add value under all conditions, but performed well with slow-escalating outbreaks qualitatively similar to the one used in our study [39]. In our particular real-world case, we found SARIMA to be more sensitive than EARS at discovering signals related to the pH1N1 pandemic wave that hit Baltimore in the summer of 2009. However, there was no associated timeliness advantage to SARIMA, as the initial peak of ARI cases was large enough to be picked by both statistical approaches. Given the variability in real-world outbreak signals, a negative finding for one specific outbreak does not reduce the impetus to design ever more sensitive and specific surveillance systems.

We compared a CDA that used diagnostic codes, a staple data type for public health surveillance, to a CDA that took advantage of the EMR to cross-validate diagnostic codes with ARI symptoms documented in related clinical notes [8]. Our results extend our prior findings that EMR-enabled gains in case-detection performance can translate into faster outbreak detection [10], adding that further timeliness can be achieved by incorporating historical data. Because we had previously measured the performance of these CDAs relative to a large reference record review [8], we could augment the realism of our simulations by discounting the injected epidemic according to CDA sensitivity. This simulation framework supplied information that may prove helpful to public health practitioners: 1) insight into the achievable gains in detection timeliness could help justify investing into more complex epidemic detection approaches; 2) AMOC curves could be used to: a) adjust outbreak detection sensitivity according to a local jurisdiction’s capacity to investigate alerts; or b) to estimate how much resources should be reallocated to surveillance activities during periods of increased threat [42].

## Limitations

Our results are confined to our experimental conditions and do not represent the final evolution of our own surveillance system, as we did not optimize individual system components, such as diagnostic code groupings, text analyses, case- or outbreak-detection approaches. The CDA- and SARIMA-related gains in detection timeliness may be related to characteristics of the epidemic, to the EMR software itself as well as to peculiarities in medical practices, care documentation and reimbursement. The EMR software and the processes of care that it supports could change over time. Thus, the performance of EMR-based systems may be more prone to variations than simpler systems, such as those based on stereotyped emergency room chief-complaints. The methods described in this work lack disease specificity compared to laboratory-based methods. Nevertheless, automated methods can incorporate available laboratory results and identify outbreaks for which diagnostic tests are not available. Their comparatively low costs also allow broad populations to be continuously monitored.

### Summary

Modeling long-term historical data with time series statistical methods can shorten the time it takes for an EMR-based surveillance system to discover epidemics of ARI. The empirical approach used to evaluate alternative surveillance systems may represent a useful blueprint for the growing efforts to harness the EMR to benefit public health [43].

## Supporting information

S1 File Background and injection data. Excel spreadsheet containing daily case counts for s17, s33, CDA1, CDA2, CDA3, injections for s17, s33, and the injection issued by the mathematical epidemic model.
(XLSX)

S2 File Epidemic model. The file is a compressed folder containing instructions and all files required to run and adjust the epidemic model used in this work.
(ZIP)
