# Supplementary material for: Can long-term historical data from electronic medical records improve surveillance for epidemics of acute respiratory infections? A systematic evaluation
Source: PLoS One. 2018 Jan 31;13(1):e0191324. doi: 10.1371/journal.pone.0191324 (PMC5791979; doi:10.1371/journal.pone.0191324)
Supplement: S2 File — The file is a compressed folder containing instructions and all files required to run and adjust the epidemic model used in this work. (ZIP) [file pone.0191324.s002.zip › Outbreak Generator copy/Instructions.pdf]

THE OUTBREAK GENERATOR

(You will need MATLAB version 7)

Create a folder called Results.
Follow the steps below in order. After each step, save the results files to the Results folder (ie back up), save the relevant results files to the next model folder AND DELETE the results files in the model folder whose MATLAB file you have just run.

Epidemic Model

1. Create a folder called "Epidemic Model"
2. Save *Epidemic_5a.m*, the file that solves the equations, and *Zip_Code_Densities.xls*, the required data file, in the folder called "Epidemic Model".
3. Open MATLAB and set the current directory to "Epidemic Model".
4. Open *Epidemic_5a.m* and run the program (F5 key or the "Run" icon).
5. The program generates 30 potential epidemics (one for each zip code) and stores each one separately in a series of excel files called *21201.xls*, *21202.xls*....and so on.

Spatial Model

1. Create a folder called "Spatial Model"
2. Place *Zip_Code_Model5.m*, the file that implements the stochastic spatial model, into the folder called "Spatial Model". Place *Zip_Code_Densities.xls*, *Zip_Code_Distances.xls*, *Prob_of_Intro.xls* and each of the 30 Excel epidemic files generated by *Epidemic_5.m*. into the same folder
3. Open MATLAB and set the current directory to "Spatial Model".
4. Open *Zip_Code_Model5.m* and run the program (F5 key or the "Run" icon).
5. The program generates a Excel results file called "*Sorted_Results.xls*"

Case Generator

1 Create a folder called "Case Generator"
2 Place *Case_Generator5.m*, the file that implements the case generator into the folder called "Case Generator". Place *Prob_of_Discovery.xls* and *Sorted_Results.xls* into the same folder
3 Open MATLAB and set the current directory to "Case Generator".
4 Open *Case_Generator5.m* and run the program (F5 key or the "Run" icon).
5 The program generates Excel results files called "*Discovered_Cases.xls*", *"VA_Recorded_Cases.xls*", and *"Dataset.xls*".

Date Generator

1. Create a folder called "Date Generator"
2. Place *Date_Generator5a.m*, the file that implements the case generator into the folder called "Case Generator". Place *Dataset.xls* into the same folder
3. Open MATLAB and set the current directory to "Date Generator".
4. Open *Datse_Generator5a.m* and run the program (F5 key or the "Run" icon).
5. The program generates an Excel results file called "*Jills_Data.xls".*

Note: there *will* be software bugs. At the moment, all looks to be running properly, but we will not know for sure until we have run this sequence of programs many times. Please let me know if you see any odd or counterintuitive result.
