# Supplementary material for: Can long-term historical data from electronic medical records improve surveillance for epidemics of acute respiratory infections? A systematic evaluation
Source: PLoS One. 2018 Jan 31;13(1):e0191324. doi: 10.1371/journal.pone.0191324 (PMC5791979; doi:10.1371/journal.pone.0191324)
Supplement: S2 File — The file is a compressed folder containing instructions and all files required to run and adjust the epidemic model used in this work. (ZIP) [file pone.0191324.s002.zip › Outbreak Generator copy/Outbreak Generator Documentation.pdf]

Syndromic Surveillance System: The Outbreak Generator

Report Dated August 18th, 2005

Gary Smith, MA, MA, D.Phil.
105 Kennett Ridge Drive,
Kennett Square.
PA 19348.

garys@vet.upenn.edu
(610) 444 3129
(610) 563 5942

Submitted to:

Trish M. Perl, MD, MSc
Associate Professor of Medicine and Pathology
Hospital Epidemiologist
Johns Hopkins Medical Institutions
425 Osler, 600 North Wolfe Street
The Johns Hopkins Hospital
Baltimore, MD 21287

Sylvain DeLisle MD, MBA
Associate Professor of Medicine
University of Maryland, School of Medicine
Associate Chief of Staff, Performance Improvement
VA Maryland Health Care System
5D-138 Baltimore VA Medical Center
10 N Greene St
Baltimore MD 21201

1. AIM

To simulate a epidemic of an influenza-like illness (ILI) in the Greater Baltimore area.

Our purpose is to use this simulated epidemic to generate a clinically plausible set of hospital cases whose pattern of occurrence and clinical features (if real) would warrant an immediate escalation in the public health response (eg new epidemiologic investigation, new measures for prevention and control, etc).

We note, in passing, that if the cases generated are so clinically implausible that they can be detected on that basis alone then we have failed. If on the other hand, the generated cases are sufficiently clinically plausible to escape detection on that basis alone, then we have accomplished the first step in our effort to provide a challenging substrate for the Syndromic Surveillance System (SSS) detection and alert algorithms developed in the context of the Baltimore area Veterans Administration Medical Record System.

We shall use the Outbreak Generator to:

- Establish the best compromise between sensitivity, detection delay and probability of a false alarm
- To determine how the SSS outbreak detector should be re-calibrated
- To evaluate the marginal benefit of increasing the complexity of the SSS.

2. OBJECTIVES

To develop a fully aged structured model for an epidemic illness with a latent period, infectious period and case mortality similar to those seen in during influenza outbreaks. The model should be based on a susceptible population with a size and age structure like that of Baltimore and should reflect recent age-specific birth and death rates in the susceptible population.

The model should provide output concerning temporal changes in the fraction of each demographic age-class that are 'susceptible', 'infected/not yet infectious', 'infectious' and 'recovered/immune'.

The model should provide information on the spatial spread of infection from the original locus and take realistic account of the processes that influence what fraction of all cases is actually recognized by the Veterans Administration Hospital System.

Finally, the results file should be in Excel format with three data columns (VISIT_DATE, ILI_COUNT, ZIP_CODE) so that the simulated data can be seamlessly inserted into the actual case records used to test the SARIMA, SatScan, and Wavelet methodologies current being used to detect outbreaks.

3. EXECUTIVE SUMMARY

The following report describes a mathematical model for an influenza-like-illness that achieves each of the objectives set out above. The model is implemented using four MATLAB "m_files" (created using MATLAB, version 7.0.4, *The Mathworks Inc*.).

The epidemic model (Epidemic_5.m): On execution, this file reads an Excel spreadsheet called Zip_Code_Densities.xls, which contains 30 zip code numbers in column 1 and the number of people resident in that zip code in column 3; it then generates a deterministic, age-structured epidemic outbreak for each zip code. Given the model parameters, the course of the outbreak is determined entirely by the initial conditions (primarily, the density of susceptible hosts). The results for each zip code are saved to a separate Excel file. The Excel file names consist of the zip code number and the Excel file extension (eg 21201.xls). Columns 1-20 of the Excel file contain the number of new cases in each five-year age-class. Column 21 is the zip code. Column 22 is the total number of infectious people in that zip code in that week. Column 23 is the week of the outbreak in that zip code (beginning at week 0 and ending at week 53).

The spatial model (Zip_Code_Model5.m): On execution, this file reads Zip_Code_Densities.xls, Zip_Code_Distances.xls, Prob_of_Intro.xls and each of the Excel results files generated by *Epidemic_5.m*. Zip_Code_Distances.xls is currently a 30x30 matrix containing the distance (in miles) between each pair of zip codes. Prob_of_Intro.xls is currently a 1x30 matrix containing the number 1 in that cell corresponding to the zip code in which the epidemic begins (we can use this file to configure all manner of initial infection scenarios, including viral plumes). *Zip_Code_Model5.m* generates the spatial components of the epidemic. That is, beginning with the first infected zip code(s), it calculates the likelihood that the infection will spread to other zip codes in each successive week and accumulates the epidemic data into a single sorted results file with the same format as the Excel files generated by the epidemic model. This single results file is called *Sorted_Results.xls*.

The Case Generator (Case_Generator5.m): On execution this file reads Sorted_Results.xls and Prob_of_Discovery.xls. The latter lists the probability of hospitalization, the probability of visiting an ER, and probability of visiting a clinic conditional on being a case of a specified age. The purpose of the case generator is to determine what fraction of all new cases becomes known to the medical system generally, and then, what fraction of these discovered cases becomes known to the VAMHCS in particular. Several results files are generated for book-keeping reasons. Only one results file is relevant: Dataset.xls. This file consists of three columns: column 1is the number of new cases that become known to the VAMHCS; column 2 is the week number; column 3 is the zip code.

*The Date Generator (Date_Generator5**a**.m):* On execution, this file reads Dataset.xls, takes the weekly report of new cases, and assigns each case to a particular recording day for the week just

elapsed. It then sums all the new case records in a given zip code assigned to a particular day and creates a final results file. The final results file is called Jills_Data.xls and consists of three columns (column 1 is date in Excel date number format, column 2 is the ILI count, column 3 is the zip code). This is the simulated epidemic data used to test the SSS.

4. INTRODUCTION

The task was to have created, by the end of Year 2, a simulated epidemic of an ILI in the Greater Baltimore Area that could be used to explore the SARIMA, SatScan and Wavelet analyses that were currently the basis of the Outbreak Detector.

Consultations revealed that the best format for the required data file would be an Excel spreadsheet containing three columns headed “VISIT_DATE”, “ILI_COUNT” and ZIP_CODE” respectively. The column labeled, “ILI_COUNT”, would contain the number of new simulated cases of the influenza-like illness that came to the attention of the VAMHCS on the visit date recorded in column 1. The zip code recorded in column 3 referred to the home address of each recorded case. Thus,

VISIT_DATE	ILI_COUNT	ZIP_CODE
1/20/2003	17	21222
1/20/2003	21	21223

indicates that 17 new cases from zip code 21222 were recorded on January 20th, 2003. The file containing these results would be called, Jills_Data.xls. What follows shows how this file was created.

5. THE BASIC ASSUMPTIONS

Why should we use simulated epidemics to seed the database?

Methods for testing syndromic surveillance systems often use stereo-typical and highly artificial epidemic patterns to seed the database substrate of the detection algorithm (eg Reis *et al.*, 2003). This very useful in refining the basic parameters of the detection algorithm(s) but there is no reason to suppose that such stereo-typical patterns represent what will actually be recorded by the database should an epidemic event of interest occur. For example, hospital records of infectious disease, will reflect not just the current prevalence of infection, but all those other parameters that determine the morbidity associated with infection and the likelihood that a severely ill case will come to the attention of any of the institutions represented in that particular database. In other words, only a small fraction of all cases will be recorded in any given data base and there is no reason to believe that the temporal and demographic characteristics of the discovered and recorded cases will track the temporal and demographic characteristics of the outbreak as a whole (discovered and undiscovered cases combined). Because the purpose of a syndromic surveillance system is to detect real outbreaks as early as possible, it seems reasonable to develop and test the detection algorithm(s) using data seeds that reflect as nearly as possible

what would actually be recorded should an epidemic event of interest occur.

An influenza-like illness recorded in the VAMHCS data base is an ideal place to start: we have good, quantitative information concerning the risk factors for hospitalization, emergency room visits and physician consults for influenza cases - and the eligibility criteria for VAMHCS are relatively straightforward functions of susceptible population age structure and gender for any given area.

Why did we choose the model framework we did?

1. Temporal models

All good models are built to some purpose. In this case, we needed to generate a data seed that represented the number of new cases recorded by the VAMHCS each day. The cases needed to be sorted by zip code of residence and so required model had to have a spatial component. The very simplest epidemic models make the assumption that there is homogeneous mixing of susceptible and infected hosts (the "mass action" principle)(Smith and Scott, 1994). Such models necessarily have no spatial component because contacts between susceptible and infected hosts are deemed to occur as if the mixing were instantaneous. This useful family of models has a very long pedigree (Anderson and May, 1991). However, recent experience of emerging infections (HIV/AIDS, SARS, BSE, FMD) has demonstrated the value of spatial models that take explicit account of contact patterns or the "Who Mixes with Whom" matrix (eg Woolhouse and Donaldson, 2001; Keeling *et al.*, 2001; Ferguson *et al.,* 2001a, 2001b; Keeling *et al.*, 2003; Morris *et al.*, 2001). However, it remains extremely difficult to describe *and use* actual contact patterns (Edmunds *et al.*, 1997) and, often, one must use some hybrid method of representing the spatial aspects of the spread of infection. A conceptually straightforward way of doing this is to separate the population of hosts into smaller sub populations each with their own independent dynamics but including some transmission (or "coupling") between them (Keeling, 1997; Keeling *et al.*, 2004). Models using this concept are called "metapopulation" or "patch" models.

2. Spatial (metapopulation) models

Metapopulation models have been important in theoretical ecology and conservation biology for many years, and the last decade or so has seen a sharp increase in papers elaborating metapopulation dynamics (Gilpin and Hanski, 1991; Kanski, 1999, Dobson, 2003). Originally intended as a framework within which to investigate the mechanisms that foster or impede the persistence of wild animal and plant populations that existed in discrete patches, metapopulation dynamics has been co-opted by research workers interested in the persistence of infectious and parasitic diseases (eg Grenfell and Hardwood, 1997; Keeling and Gilligan, 2000)

In recent years there have been attempts to apply metapopulation dynamics models to populations that do not exist in discrete patches (Thomas and Kunin, 1999). In such cases the patches are arbitrarily delimited as an array of quadrats, for example - *or zip codes*. While there still legitimate debate about the validity of that device (Freckleton and Watkinson, 2002), it has

been successfully used to provide insights into the processes that account for changes in infectious disease occurrence in both time and space (eg Grenfell and Harwood , 1997; Keeling and Gilligan, 2000) and, most recently, to examine contiguous cull control strategies ("neighborhood control policies") for controlling the spread of directly transmitted animal diseases like FMD (Matthews et al., 2003).

Accordingly, a metapopulation model approach was adopted here.

How is the metapopulation model organized?

We selected 30 contiguous, populated zip codes in the Greater Baltimore area as the 'patches" that contained our 30 sub populations of wholly susceptible hosts (there are many more, "non-populated" zip codes in the same region that serve merely as PO BOX addresses).

We assumed that the dynamics of the infection in each zip code were independent of the dynamics of the infection in the other zip codes and could be adequately represented by a deterministic age-structured model. Given the model parameters, the course of the outbreak was determined entirely by the initial conditions (primarily, the density of susceptible hosts in the zip code of interest).

The probability of transmission between zip-codes was assumed to be determined by the distance between zip codes and the number of infectious hosts in the already affected zip codes. The respective probabilities were recalculated at each iterative step, (because the number of infectious individuals had changed, even if the distances between zip codes had not). The newly calculated probabilities provided the basis for a Monte Carlo simulation, which determined whether or not the infection was introduced to an unaffected zip code before the next iteration.

Thus, transmission within zip codes was a deterministic process entirely dependent upon the initial conditions, whereas transmission between zip codes was a stochastic process with an ever-changing probability of occurrence. For this reason, even simulations with the same initial conditions will produce different results. Nor is it to be expected that every zip code will be involved in the outbreak every time (stochastic fade-out).

How do we define a case?

We define a *discovered case* as any individual with influenza that comes to the attention of the medical services in Baltimore as the result of hospitalization, an emergency room visit, or phone call or visit to a general practitioner or equivalent clinic.

We define a *recorded case* as any in that subset of discovered cases that is recorded in the VAMHCS.

Every individual in the "infectious" class generated by the model is a potential *discovered*

case.

Several studies have shown that age is a very strong predictor of hospitalization in influenza cases (Sprenger *et al.*, 1993; Irwin *et al.,* 2001; Thompson *et al.*, 2004, Hak *et al.*, 2004) and Menec *et al.*, have also shown that the probability of visiting an emergency room or contacting a general practitioner is related to the age of the infected individual. While we are aware that there are other risk facts for each of these events (eg pre-existing malignancy, chronic heart disease, chronic respiratory disease and diabetes: Sprenger *et al.*, 1993; Irwin *et al.,* 2001; Thompson *et al.*, 2004, Hak *et al.*, 2004), we ignore this complication and assume, simply, that age is a carrier variable for all the things that matter and that, for the purposes of this simulation, it is possible to estimate how many of the infectious individuals become *discovered cases* based simply upon the age-structure of the infectious population.

Only some of the *discovered cases* will be eligible for medical services provided the VAMHCS. All of the eligible, *discovered cases* are assumed to become *recorded cases*. (This is an over estimate, of course, because not all eligible cases actually take advantage of the services offered). Eligibility is easily estimated by applying the demographic information posted on the Veteran's Administration web site for Baltimore County.

6. THE EPIDEMIC MODEL (see also Appendix A)

Overview

The potential epidemic that could be experienced by any given zip code in the model is generated by implementing the MATLAB —file, *Epidemic_5.m*. On execution, this file reads an Excel spreadsheet called *Zip_Code_Densities.xls*, which contains 30 zip code numbers in column 1, a code number (1-30) in column 2, and the number of people resident in each zip code in column 3; it then generates a deterministic, age-structured epidemic outbreak for each zip code. Given the model parameters, the course of the outbreak is determined entirely by the initial conditions (primarily, the density of susceptible hosts). The results for each zip code are saved to a separate Excel file. The Excel file names consist of the zip code number and the Excel file extension (eg *21201.xls*). Columns 1-20 of the Excel file contain the number of new cases in each five-year age-class. Column 21 is the zip code. Column 22 is the total number of infectious people in that zip code in that week. Column 23 is the week of the outbreak in that zip code (beginning at week 0 and ending at week 53).

The mathematical details

The age-structured, deterministic epidemic model for each zip code consists of a series of partial differential equations that follow the classic format (McLean and Anderson, 1988).

$$\frac{\partial X}{\partial a}+\frac{\partial X}{\partial t}=-(\lambda(a,t)+\mu(a))X(a,t)$$
$$\frac{\partial H}{\partial a}+\frac{\partial H}{\partial t}=\lambda(a,t)X(a,t)-(\delta+\mu(a))H(a,t)$$
$$\frac{\partial Y}{\partial a}+\frac{\partial Y}{\partial t}=\delta H(a,t)-(\gamma+\alpha(a)+\mu(a))Y(a,t)$$
$$\frac{\partial Z}{\partial a}+\frac{\partial Z}{\partial t}=\gamma Y(a,t)-\mu(a,t)Z$$

where X is the number of susceptible people, H is the number of people that are infected but not yet infectious, Y is the number of infectious people, and Z is the number of people who are recovered and immune (immunity is assumed to last for at least the course of the epidemic). The latent period ($1/\delta$) is one week, the infectious period ($1/\gamma$) is two weeks (both constants) and the age-dependent disease-attributable death rate $\alpha(a)$ is greatest in the very young and the very old (see below). It is assumed that the force of infection (λ) varies only with the number of infectious individuals; there are no age dependent variations in susceptibility, infectiousness, exposure or contact rates.

The demography of the population within each zip code

The age structure of the population was divided into 20 demographic age-classes (age class 1 = 0-4 years, age-class 2 - 5-9 years and so on). The proportion of the susceptible population initially in each 5 year age class was taken to be the same as that described in the 2000 census for Baltimore (www.censusScope.org. Social Science Data Analysis Network, University of Michigan)

The probability of death (not attributable to the infection) in each demographic age class was calculated from data in a table of United States Life Table Functions for the Calendar Year 1994 (Security Online, Actuarial Resources, Office of the Chief Actuary, February 4th, 2003: www.ssa.goc/OACT/index.html)

The birth rate for each demographic age-class was obtained from Guer *et al.* (1999) who summarized the US vital statistics for 1998 (their Table 3).

The additional mortality attributable to infection (α)

The overall case-mortality for influenza is reckoned to be of order 0.02% (Bell & Davis. 2001). However, although numerous authorities agree that the case mortality is greatest in the very young and very old age-classes there are few published data that report other than excess

deaths during the influenza season. In order to estimate the required age-specific probability of dying (conditional on being a case) one needs to have data on the actual age-specific incidence of disease as well as some estimate of under-reporting. Such analyses are hard to come by. Mamalund's (2001) analysis of influenza in ethnic minorities in Norway provides a useful starting point and although it is not precisely what is required, the data in his Figure 2 provide the basis for the age-specific probabilities of death attributable to infection that are recorded in the MATLAB file. It should be noticed, though, that the deaths that result from using this functional form are almost an order of magnitude higher than that expected in anything other than infection with a highly pathogenic strain.

Using the MATLAB file

The equations are solved using Euler's method. The MATLAB file that solves the equations for each zip-code in turn is called *Epidemic_5.m* (case-sensitive). The file can be invoked by pressing the F5 key.

The MATLAB file is filled with log notes and comments to enable the user to understand the logic (mathematical and biological) and some of the history of model development. Certain parts of the code have been rendered temporarily unusable by the insertion of comment marks (%). Such code is not required by the current implementation but might be useful in future versions and has been "moth-balled".

The procedure is this.

- Create a folder called "Epidemic Model"
- Save *Epidemic_5.m*, the file that solves the equations, and *Zip_Code_Densities.xls*, the required data file, in the folder called "Epidemic Model".
- Open MATLAB and set the current directory to "Epidemic Model".
- Open *Epidemic_5.m* and run the program (F5 key or the "Run" icon).
- The program generates 30 potential epidemics (one for each zip code) and stores each one separately in a series of excel files called *21201.xls*, *21202.xls*....and so on.

The more esoteric features of the epidemic model have been hustled away into Appendix A.

7. THE SPATIAL MODEL (see also Appendix B)

Overview

The Epidemic model has created a *potential* epidemic for each of the 30 zip codes in the Greater Baltimore area. These outbreaks are designated "potential" epidemics because there is no certainty that the infection will be introduced into each and every one of the 30 zip codes. The risk of introduction depends upon the distance from already affected zip codes and the number of

infectious people in those zip codes. Column 23 of each of the epidemic files (eg *21201.xls*) contains a week number which begins at 0 and terminates at 52. Thus the "Week Number" in these files represents the elapsed time since the infection was first introduced into that zip code, but not a date. We need to

- define which zip codes were infected initially (at "Week Number" 0)
- determine the "Week Number" (relative to the start of the epidemic) upon which the remaining zip codes became infected (if at all) and alter successive "Week Numbers" accordingly, and
- combine the data for all the involved zip codes into a single results file.

This is the purpose of the MATLAB file called *Zip_Code_Model5.m* (case sensitive). On execution, this file reads *Zip_Code_Densities.xls*, *Zip_Code_Distances.xls*, *Prob_of_Intro.xls* and each of the 30 Excel epidemic files generated by *Epidemic_5.m*.

Zip_Code_Distances.xls is currently a 30x30 matrix containing the distance (in miles) between each pair of zip codes. *Prob_of_Intro.xls* is currently a 1x30 matrix containing the number 1 in that cell corresponding to the zip code in which the epidemic begins (we can use this file to configure all manner of initial infection scenarios, including viral plumes).

Zip_Code_Model5.m generates the spatial components of the epidemic. That is, beginning with the first infected zip code(s), it calculates the likelihood that the infection will spread to other zip codes in each successive week and accumulates the epidemic data into a single sorted results file with the same format as the Excel files generated by the epidemic model. This single, results file is called *Sorted_Results.xls.*

The probability that the infection will be introduced into a given zip code

The main program loop *Zip_Code_Model5.m* works in weekly iterations. Each week, the program calculates anew[1] the probability (π_{zipij}) that zip code i will be infected by zip code j and then uses Monte Carlo simulation methods to decide whether infection actually occurs based upon the calculated probability.

The probability (π_{zipij}) that zipcode i will be infected by zip code j is assumed to depend upon

- the distance (D in miles) from zip code j, and
- the number of infectious people (Y) in zip code j.

We use a logistic function in standard manner to calculate this probability (the logistic function

---

[1] The probability (π_{zipij}) depends upon two things: the distance between zip codes and the number of infectious people. Distance does not change, but the number of infectious people increases at first and then declines. It is the change in the number of infections people that requires us to recalculate the probabilities each week.

necessarily constrains the value of π_{zipij} such that $0 \le \pi_{zipij} \le 1$). Thus

$$\pi_{zipij} = 1 - \frac{1}{1+\exp(-a_2 - b_2 D)}$$

where

$$a_2 = \frac{c_1}{1+\exp(-a_1 - b_1 Y)}$$

The constants a_1, b_1, b_2, and c_1 are arbitrary and selected using iterative methods (guess and check) to create the velocity and extent of spatial spread required for each simulation.

Current values are $a_1 = -5$, $b_1 = 0.01$, $b_2 = 0.5$, $c_{1\,=} = -8$.

The Monte Carlo Simulation

The value of π_{zipij} for each zip code pair is calculated at the beginning of each weekly iteration of the program. The program takes each uninfected zip code in turn, reads each successive value of π_{zipij} for that zip code and generates a random number between 0 and 1. If the number is less than the current value of π_{zipij}, then the zip code is deemed to have become infected. If the number is not smaller than the current value of π_{zipij} for that zip code pairing, then the zip code is not deemed to have become infected. Multiple introductions into a given zip code are ignored. Only the first matters. This is a necessary consequence of the assumption that the epidemics within zip codes are straightforwardly deterministic.

At each iteration, the epidemic data for the newly infected zip codes are added into the results file and the process is repeated (up to 52 times).

How do we handle "Week Numbers"?

The main loop in *Zip_Code_Model5.m* updates the situation on a weekly basis. At each iteration we calculate the probability that an uninfected zipcode could be infected by some other and then use the MATLAB "rand" function to determine whether infection actually occurs or not (see Monte Carlo simulation above). If a zip code is infected, the program reads the epidemic data file for this zipcode and then alter the values in the "time" column (column 23) to reflect the time when the infection was first introduced into this zipcode. Thus, the "time" column in the original epidemic files contains a "Week Number" that begins at 0 and terminates at 52. These "Week Numbers" are altered by *Zip_Code_Model5.m* as each zip code joins the epidemic such that the first "Week Number" for the epidemic in that zip code now reflects the "Week Number" that the infection was first introduced.

The results file: Sorted_Results.xls

Zip_Code_Model5.m creates a combined Excel results file called *Sorted_Results.xls*. This file includes data from all the zip codes that joined the epidemic (which will not necessarily be all 30 zip codes because of the stochastic nature of the spatial model). The data are sorted first by "Week Number" and then by "Zip Code" (Columns 23 and 21 respectively). Columns 1-20 in *Sorted_Results.xls* report the number of new infections in each five year age class, and column 22 reports the total current number of infectious people.

Using the MATLAB file

Zip_Code_Model5.m can be invoked by pressing the F5 key.

The MATLAB file is filled with log notes and comments to enable the user to understand the logic (mathematical and biological) and some of the history of model development. Certain parts of the code have been rendered temporarily unusable by the insertion of comment marks (%). Such code is not required by the current implementation but might be useful in future versions and has been "moth-balled".

The procedure is this.

- Create a folder called "Spatial Model"
- Place *Zip_Code_Model5.m*, the file that implements the stochastic spatial model, into the folder called "Spatial Model". Place *Zip_Code_Densities.xls*, *Zip_Code_Distances.xls*, *Prob_of_Intro.xls* and each of the 30 Excel epidemic files generated by *Epidemic_5.m*. into the same folder
- Open MATLAB and set the current directory to "Spatial Model".
- Open *Zip_Code_Model5.m* and run the program (F5 key or the "Run" icon).
- The program generates a Excel results file called "*Sorted_Results.xls*"

THE CASE GENERATOR (see also Appendix C)

Overview

Having implemented both the Epidemic model and the Spatial model we have an Excel file called *"Sorted _Results.xls"*. Columns 1-20 in this file contain the number of new infections in each five-year age-class arranged by "Week Number" and "Zip Code". Not all infections come to the attention of the medical authorities; those that do we shall call "Discovered Cases". Those that are recorded in the VAMHCS we shall call "Recorded Cases". We need to

- calculate the number of new infections that become "Discovered Cases", and
- calculate the number of "Discovered Cases: that become "Recorded Cases"

This is the purpose of the MATLAB file called *Case_Generator5.m*. On execution this file reads

a data file called *Prob_of_Discovery.xls* (a table showing the probability of hospitalization, the probability of visiting an ER, and probability of visiting a clinic conditional on being a case of a specified age), and *Sorted_Results.xls* (the file created by the Spatial model). The Case Generator uses Monte Carlo methods to determine what fraction of all new infections become known to the medical system generally, and then, what fraction of these discovered cases becomes known to the VAMHCS in particular. Several results files are generated for book-keeping reasons. These include *Discovered_Cases.xls*, which contains all Discovered Cases grouped by five-year age-class, week number and zip code (columns 1-20: cases by age-group, column 21: zip code; column 22: total number of infectious people, column 23: week number), and *VA_Recorded_Cases.xls*, which contains all Recorded Cases in VHNHCS data base grouped by five-year age-class, week number and zip code (columns 1-20: cases by age-group, column 21:zip code; column 22: total number of infectious people , column 23: week number).

The principal results file is called *Dataset.xls.* This file consists of three columns: column 1is the number of new cases that become known to the VAMHCS; column 2 is the week number; column 3 is the zip code.

How do we calculate the number of Discovered Cases from the number of new infections?

Menec *et al.* (2003) measured the increased risk of hospitalization, of emergency room visits and of visits to a physician associated with the influenza season in Winnipeg, Canada between 1995-1999. The increased risk was measured as annualized excess rates per 100,000 age-specific population and was age dependent. It was possible to use these data to derive the conditional probability of each of these events for our Baltimore population given certain assumptions about the absolute of at least one of the probabilities. Specifically, we needed to decide what value we were going to assign the probability of hospitalization, all other probabilities could then be calculated relative to this using the data provided by Menec *et al.* (2003). In fact it does not matter what precise value we choose because altering this value is one way of modulating the "severity" of the simulated epidemic. However, in this preliminary phase, we chose a value that corresponds with current experience of influenza.

In fact, it turns out to be very difficult to estimate how many influenza cases involve hospitalization because influenza infections typically are not confirmed virologically or specified on hospital discharge forms (Thompson et al., 2003a). However, Irwin *et al.* (2001) estimated that two percent of influenza/ILI episodes involve hospitalization (ILI cases were identified in an automated claims database on the basis of associated ICD-9 codes). This was the value we chose. The details of the calculation that were based upon this assumption and that led to the probabilities listed in *Prob_of_Discovery.xls* can be found in Appendix C.

The probabilities contained in *Prob_of_Discovery.xls* vary with age. *Case_Generator5.m* takes each new case listed in *Sorted_Results.xls* and looks up the age appropriate probability of hospitalization. *Case_Generator5.m* generates a random number between 0 and 1 and if the number is less than the age-appropriate probability for that infection the case is deemed to be

hospitalized and thus a "Discovered Case". If the random number is greater than the age-appropriate probability for that infection, then the process is repeated using first, the age-appropriate probability of visiting an emergency room, and second, the age-appropriate probability of contacting a general practitioner. At the end of this sequence, the new infection has either been re-designated a "Discovered Case" or deleted. The procedure is repeated for every new infection in turn.

How do we calculate the number of Recorded Cases from the number of Discovered Cases?

The Department of Veteran's Affairs website publishes statistics for the proportion of the population that is eligible for medical care under Veteran's Administration. For example, in Baltimore County, 0.19 of males 18-64 years old are eligible for care and so are 0.71 males older than 65. (http://www.va.gov/vetdata/Census2000/index.htm). The corresponding figures for females are 0.013 females 18-64 years old, and 0.016 females older than 65. We also note that patients in the age group, 0-19 years, constitute less than 0.2% of the entire VAMHCS patient population whereas the same age group is 18% of the general hospital population. It seems not unreasonable to assume that simulated cases in patients less than 20 years old will NOT come to the attention of the VAMHCS. We used these age- and gender appropriate probabilities in conjunction with a Monte Carlo process like that described immediately above to calculate the number of Discovered Cases that were also Recorded Cases.

Using the MATLAB file

Case_Generator5.m can be invoked by pressing the F5 key.

The MATLAB file is filled with log notes and comments to enable the user to understand the logic (mathematical and biological) and some of the history of model development. Certain parts of the code have been rendered temporarily unusable by the insertion of comment marks (%). Such code is not required by the current implementation but might be useful in future versions and has been "moth-balled".

The procedure is this.

- Create a folder called "Case Generator"
- Place *Case_Generator5.m*, the file that implements the case generator into the folder called "Case Generator". Place *Prob_of _Discovery.xls* and *Sorted_Results.xls* into the same folder
- Open MATLAB and set the current directory to "Case Generator".
- Open *Case_Generator5.m* and run the program (F5 key or the "Run" icon).
- The program generates Excel results files called "*Discovered_Cases.xls*", *"VA_Recorded_Cases.xls*", and *"Dataset.xls*".

THE DATE GENERATOR (see also Appendix D)

Overview

The results file, *Datasets.xls* , produced by Case Generator, has three columns: column 1is the number of new cases that have become known to the VAMHCS over the previous week (the "Recorded Cases"); column 2 is the week number; column 3 is the zip code.

The final step in creating the simulated epidemic is to take the results in *Datasets.xls* and create a new Excel file in a format consistent with the VAMHCS data base. The file will contain three columns: column 1 is the Visit Date, column 2 is the ILI Count, column 3 is the Zip Code. We need to

- decide the date on which the epidemic began (ie the date when the infections were introduced... equivalent to Week Number 0)
- assign a Visit Date to each Recorded Case and convert the date to Excel format
- sum and sort all recorded cases with the same Visit Date by Zip Code, and
- rearrange the columns in Dataset.xls so that they match the required order.

This is the purpose of the MATLAB file called, *Date_Generator5**a**.m*. On execution, *Date_Generator5**a**.m* reads *Dataset.xls*, takes the weekly report of new Recorded Cases, and assigns each case to a particular Visit Date in the week just elapsed. It then sums all the new case records in a given Zip Code assigned to a particular Visit Date and creates a final results file. The final results file is called *Jills_Data.xls* and consists of three columns (column 1 is the Visit Date in Excel date number format, column 2 is the ILI count, column 3 is the Zip Code). This is the simulated epidemic data used to test the SSS.

How do we tell the Date Generator when the epidemic began?

Line 32 of the MATLAB file, *Date_Generator5**a**.m*, is as follows:

```
origin = '1-oct-2002'; % Day zero for the epidemic; alter as needed.
```

This indicates that the epidemic began on October 1st, 2002. If we wished the epidemic to begin on July 1st, 2003, we would use the MATLAB editor to rewrite this line:

```
origin = '1-jul-2003'; % Day zero for the epidemic; alter as needed.
```

We would then save the file and run the program.

How do we assign a Visit Date to each Recorded Case?

Modelers often complain that one of the biggest difficulties they face in estimating the duration of an incubation period (for example) is the variability of the so-called reporting delay (the time between the onset of symptoms and the date upon which the existence of the case becomes known to medical or veterinary authorities). One consequence of the reporting delay is that, over short time periods, the order in which cases are recorded is not necessarily the order in which they occurred (Donnelly *et al.*, 1997). We exploit this usually irritating phenomenon in the method by which we assign a Visit Date to each Recorded Case.

The Epidemic model generates infections on a weekly basis, which keeps computational time down to a few seconds. The Spatial model and the Case Generator also deal with infections and cases on a weekly basis. However, the VAMHCS data base records cases on a daily basis. The Date Generator is a device that obviates the need for the Epidemic model to generate infections on a daily basis (with a consequent increase in computational time). In short, *Date_Generator5**a**.m* takes each of the Recorded Cases with the same Week Number and randomly assigns each one a Visit Date sometime in the previous week.

The first step in the process is to convert all Week Numbers to dates in Excel serial number format relative to the date upon which the epidemic began. Next, the program takes each of the Recorded Cases in turn, generates a random number between 0 and 6, and subtracts this number from the corresponding Excel serial number in the same row. The result is the Excel serial number corresponding to the Visit Date for that Recorded Case.

Using the MATLAB file

Date_*.m* can be invoked by pressing the F5 key.

The MATLAB file is filled with log notes and comments to enable the user to understand the logic (mathematical and biological) and some of the history of model development. Certain parts of the code have been rendered temporarily unusable by the insertion of comment marks (%). Such code is not required by the current implementation but might be useful in future versions and has been "moth-balled".

The procedure is this.

- Create a folder called "Date Generator"
- Place *Date_Generator5a.m*, the file that implements the case generator into the folder called "Case Generator". Place *Dataset.xls* into the same folder
- Open MATLAB and set the current directory to "Date Generator".
- Open *Datse_Generator5a.m* and run the program (F5 key or the "Run" icon).
- The program generates an Excel results file called "*Jills_Data.xls"*.

A note on the recommended file structure for running the four components of the model

It may seem rather tedious to create four separate folders (Epidemic model, Spatial Model, Case generator, Date generator) and be forever copying files from one to the other, but experience shows that this method helps to avoid errors. It is very important that the run be carried out in sequence (Epidemic model → Spatial Model → Case generator → Date generator) and completed before rerunning, say, *Epidemic_5.m* again. If *Epidemic_5.m* is run before the sequence is completed, the program will over write the previous results files and chaos ensues...

The best method is

- to run the complete sequence, and
- then to copy and rename *all* the results files before running the sequence again.

If this is not done, it is possible to get overwritten files that contain elements of previous runs. This arises because *Sorted_Results.xls* (for example) is a different length from run to run. If a previous, long undeleted version of *Sorted_Results.xls* is overwritten by a short, current version of *Sorted_Results.xls,* the last few lines of the previous version will appear at the end of the current version.

Appendix A. The Epidemic Model

Epidemic_5.m requires just one data file: *Zip_Code_Densities.xls*. This file contains 30 zip code numbers in column 1, a code number in column 2, and the number of people resident in that zip code in column 3.

Zip_Code_Densities.xls

Zip Code	Coding	Pop Dens
**21201**	1	15097
**21202**	2	23572
**21205**	3	18440
**21206**	4	50154
**21207**	5	47955
**21208**	6	31497
**21209**	7	21683
**21210**	8	11733
**21211**	9	16711
**21212**	10	35295
**21213**	11	38442
**21214**	12	21285
**21215**	13	66358
**21216**	14	36242
**21217**	15	41636
**21218**	16	55059
**21222**	17	54923
**21223**	18	31016
**21224**	19	48536
**21225**	20	31428
**21226**	21	6720
**21227**	22	33799
**21228**	23	48601
**21229**	24	49764
**21230**	25	33098
**21231**	26	15734
**21234**	27	69100
**21236**	28	15656
**21237**	29	21414
**21239**	30	28810

Epidemic_5.m creates 30 results files called *21201.xls*, *21202.xls... 21239.xls*. Columns 1-20 in these files contain the number of new infections created over the previous week in each five-year age-class; column 21 contains the zip code number; column 22 contains the number of currently

infectious individuals (this will NOT be the same as the sum of the new cases because it will include individuals accumulated from previous weeks that have not yet died or recovered); column 23 contains the Week Number (a number between 0 and 52) that reflects the elapsed time in weeks relative to the time when the initial infections were introduced into the zip code.

Columns 15- 23 of the first few rows of *21239.xls* are printed below.

0	0	0	0	0	0	21239	15.79825	0
0.767512	0.654204	0.417577	0.265954	0.051145	0.00769	21239	34.28768	1
0.745635	0.635462	0.405821	0.258043	0.05019	0.007556	21239	44.71791	2

Notice that there are 15.79 currently infectious people in the first row but there are no new infections recorded in columns 15-20. The 15.79 currently infectious people are the "introduced infections" that begin the epidemic. They are not regarded as "new infections" because they did not arise in zip code 21239.

Also notice that we unabashedly claim that there are 0.797512 new cases in age group, 70-74 years. Modelers sometimes forget that clinicians do not share our comfort level when it comes to dealing with non-integer numbers of patients. Remember, though, that deterministic models are approximations at best - good approximations when dealing with large numbers of hosts, as we are - but approximations nevertheless. The fractional patients are dealt with later on in the Case Generator by a process of "rounding down to the nearest integer".

Appendix B. The Spatial model

Zip_Code_Model5.m requires 33 data files: Zip_Code_Densities.xls (see Appendix A), *Zip_Code_Distances.xls, Prob_of_Intro.xls* and each of the Excel results files generated by *Epidemic_5.m* (ie *21201.xls*, *21202.xls... 21239.xls*)

Zip_Code_Distances.xls is currently a 30x30 matrix containing the distance (in miles) between each pair of zip codes. The first 6 rows and 6 columns are printed below.

0.0	0.5	2.2	5.1	5.6	8.1
0.5	0.0	1.7	4.6	6.1	8.5
2.2	1.7	0.0	3.2	7.6	9.6
5.1	4.6	3.2	0.0	9.4	10.4
5.6	6.1	7.6	9.4	0.0	3.7
8.1	8.5	9.6	10.4	3.7	0.0

There are no row or column labels, but the first column deals with zip code 21201, the second with 21202, the third with 21205 and so on in the order seen in Zip_Code_Densities.xls (see Appendix A). The rows are dealt with similarly. Thus the distance between the center of zip code 21206 (row 4 or column 4) and the center of zip code 21202 (row 2 or column 2) is 4.6 miles (the intersection of row 4 and column 2).

Prob_of_Intro.xls is currently a 1x30 matrix containing the number 1 in that cell corresponding to the zip code in which the epidemic begins (we can use this file to configure all manner of initial infection scenarios, including aerial plumes).

The first 6 columns of *Prob_of_Intro.xls* are printed below.

1	0	0	0	0	0

The columns correspond to the zip codes listed in Zip_Code_Densities.xls (see Appendix A). The first column deals with zip code 21201, the second with 21202, the third with 21205 and so on. There is a number 1 in column 1. Which means that the epidemic began in zip code 21201, right in the heart of Baltimore. The other columns contain zeros, which indicates that the epidemic did not begin there! If we wished the epidemic to begin with infections introduced into 21202 and 21206, we would simply edit *Prob_of_Intro.xls* as follows

0	1	0	1	0	0

Linear plume infections can be initiated by reference to a map of Baltimore zip codes and inserting the number 1 in the appropriate columns.

Zip_Code_Model5.m generates a single results file called *Sorted_Results.xls*. Columns 1-20 in this files contain the number of new infections created over the previous week in each five-year age-class; column 21 contains the zip code number; column 22 contains the number of currently infectious individuals (this will NOT be the same as the sum of the new cases because it will include individuals accumulated from previous weeks that have not yet died or recovered); column 23 contains the Week Number (a number between 0 and 52) that reflects the elapsed time in weeks relative to the time when the initial infections were introduced into the zip code.

Columns 15- 23 of the first few rows of *Sorted_Results.xls* are printed below.

0	0	0	0	0	0	0	0
0	0	0	0	0	21201	15.79825	0
0.653667	0.417234	0.265736	0.051103	0.007683	21201	34.26735	1
0	0	0	0	0	21202	15.79825	1
0	0	0	0	0	21218	15.79825	1
0	0	0	0	0	21223	15.79825	1
0	0	0	0	0	21224	15.79825	1
0.634452	0.405176	0.257632	0.05011	0.007544	21201	44.66749	2
0.654073	0.417493	0.265901	0.051134	0.007688	21202	34.2827	2
0	0	0	0	0	21210	15.79825	2
0	0	0	0	0	21211	15.79825	2
0	0	0	0	0	21212	15.79825	2
0	0	0	0	0	21215	15.79825	2

The format is identical to *21239.xls* (Appendix A), the difference being that *Sorted_Results.xls* combines the epidemic data for *all* involved zip codes into a single file. We can see that the epidemic began in Week Number 0 in zip code 21201. By the end of the Week Number 1, zip codes 21202, 21218, 21223 and 21224 had become involved. Note the epidemic data are sorted first by Week Number (column 23) and then by zip code (column 21). The number 15.79825 repeats because the epidemic is started in the same way in each zip code - with the introduction of 15.79825 infections. By the end of Week Number 2, zip code 21201, where the epidemic began, has 44.667 currently infectious individuals and 0.007544 new infections have arisen in the 95-99 year age class (see Appendix B for remarks on fractional patients).

Appendix C. The Case Generator

INTRODUCTION

This section describes how we decide which of the simulated ILI infections are hospitalized, present at an emergency room, or visit a physician (all other infections are lost to the record). The relative frequency of occurrence of these events was recently described by Menec et al., (2003) who studied the impact of ILI on the incidence of hospitalizations, physician visits and emergency room visits in Winnepeg. This study is the basis of our initial calculations, but in order to convert relative occurrence into the case-specific probabilities, we needed, first, to define an absolute probability for at least one of the events of interest (eg hospitalization).

It is difficult to estimate how many influenza cases involve hospitalization because influenza infections typically are not confirmed virologically or specified on hospital discharge forms (Thompson et al., 2003a). However, Irwin *et al.* (2001) estimated that two percent of influenza/ILI episodes involve hospitalization (ILI cases were identified in an automated claims database on the basis of associated ICD-9 codes). We shall assume that this is the baseline figure for North America.

Menec *et al.* (2003) organized their observations in terms of patient age. We note, in passing, that age is a very strong predictor of hospitalization in influenza cases, but so too are pre-existing malignancy, chronic heart disease, chronic respiratory disease and diabetes (Sprenger *et al.*, 1993; Irwin *et al.,* 2001; Thompson *et al.*, 2004, Hak *et al.*, 2004). We shall ignore this complication and assume, simply, that age is a carrier variable for all the things that matter.

THE PROBABILITY OF HOSPITALIZATION, PHYSICIAN VISITS AND VISITS TO AN EMERGENCY ROOM ASSOCIATED WITH INFLUENZA/ILI

Table 1 (estimated from data in Menec et al., 2003)

Age	Hospitalization	Emergency Room	Physician visits
0-14years	0.00214	0.00389	0.05180
15-64 years	0.00034	0.00042	0.02270
65+ years	0.00537	0.00190	0.03760

Menec *et al.* (2003) measured the increased risk of hospitalization, emergency room visits and visits to a physician associated with the influenza season in Winnipeg, Canada between 1995-1999. The increased risk was measured as annualized excess rates per 100,000 age-specific

population. These incidence rates were sufficiently small that it is justified to interpret them directly as age-specific probabilities of hospitalization, visiting an emergency room and visiting a physician during influenza season. However, since we do not know how many cases and non-cases there were in each age group we are in some difficulty with respect to estimating the probability of a particular outcome conditional on being a case.

The calculation performed by Menec et al. (2003) was as follows

$$Excessrisk = \frac{(R.p + C.\pi - R.p)}{R} = \frac{C\pi}{R}$$

where R was the population at risk of hospitalization in *each age group*, p was the baseline probability of being hospitalized (say), C was the number of influenza cases each year, and π was the probability of a case of influenza being hospitalized. This formulation assumes that being hospitalized for influenza or for some other cause are not mutually exclusive events. Menec et al., (2003) standardized the ratio, $C\pi/R$, for a population of 100,000. Notice that C/R is the attack rate (or cumulative incidence), the proportion of those at risk that became cases.

The problem is we know neither the attack rate nor the value of π. All we know is their product. If we assume that attack rate is the *same* for each age-group, the probabilities in Table 1 exactly reflect the relative risk of being hospitalized, or presenting at an emergency room or visiting a physician (conditional upon being a case), but do not provide an absolute measure of risk.

In order to get any further, we remind ourselves that we are creating simulated ILI epidemics, and that it is *useful* to be able to vary the morbidity of each epidemic. For example, if we wished to simulate an epidemic in which 2% of cases were hospitalized, the value of π averaged over all age groups would be $\pi = 0.02$. Thus, if we designate the relative risks of hospitalization in age group i shown Table 1 as M_i (where i=1to 3), we can write

$$M_i = \frac{C\pi_i}{R} = A\pi_i$$

whence

$$\pi_i = \frac{M_i}{A},$$

and so

$$0.02 = \frac{M_1 p_1}{A} + \frac{M_2 p_2}{A} + \frac{M_3 p_3}{A} = \frac{1}{A}\sum_1^3 M_i p_i,$$

where p_i is the probability of an individual being in age group, i. For Baltimore, the required values of p_i are 0.21, 0.67 and 0.12 respectively (estimated from the US census 2000). Substituting these in the above equation gives 0.02 = 0.0013/A, and so 1/A = 15.38.

Some interpretation is in order. The value of 1/A is the multiplier that would have to be applied to the probabilities in Table 1 to convert them from relative to absolute risks of the given outcome (conditional on being a case). The multiplier is simply the reciprocal of the influenza attack rate (1/A = R/C). Thus, if we accept that, on average, 2% of influenza cases are hospitalized (and further assume that the age distributions of the populations of Winnipeg and Baltimore are similar), the excess hospitalization rates recorded by Menec et al. (2003) are consistent with an average influenza attack rate of 1/15.38 = 0.065, or 6.5%). In fact, it doesn't matter whether we accept the 2% figure or not (the above argument is presented simply to demonstrate the plausibility of the calculations). What matters is that we can

- decide what proportion of our simulated cases are hospitalized (ie decide on the morbidity of the simulated case)
- use the method given above, in conjuction with the data in Table 1, to estimate the multiplier required to calculate the absolute, age dependent risks of hospitalization emergency room visits or physician visits that are consistent with our decision.

The main assumptions are

- the attack rate is independent of age
- the relative relationships shown in Table 1 continue to apply for all moderate, specified hospitalization rates. (This last assumption is important because it is possible to imagine a situation in which morbidity increases such that the *relative* frequency of hospitalization increases with respect to (say) physician visits).

To proceed further, we also assume that being hospitalized, presenting at an emergency room and visiting a physician are mutually exclusive events, at least with respect to the *first time* the case appears in the medical record. We understand that cases which first present to a physician or emergency room may eventually be hospitalized (to cite merely one permutation), but, for present purposes, we are interested in only the first appearance of the case in the medical record, not what happens subsequently. For the same reason, in the algorithm that follows, we argue it is reasonable to consider the probability of hospitalization before the probability of presenting at an emergency room, and the probability of presenting at an emergency room before the probability of visiting a physician.

The decision tree shown in Figure C1, traces the steps for just a single age group.

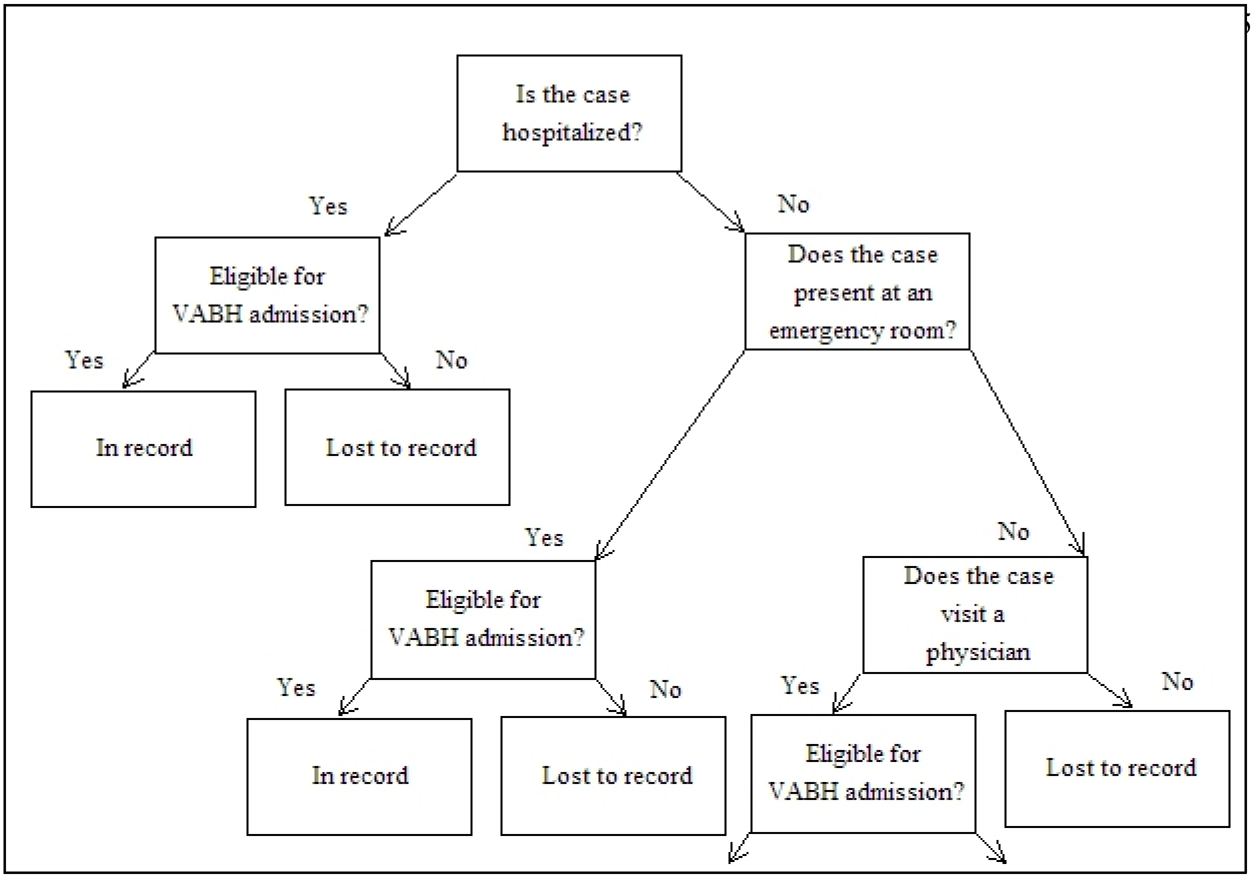

Figure C1 The decision tree used to determine whether a case is hospitalized, presents at an emergency room or visits a physician. See text for details.

However, it will be understood that the steps are identical for all age groups. Once it has been established that a case will be recorded (or not), we move to the next algorithm to determine whether it will be recorded by the VAMHCS system. .

ELIGIBILITY FOR MEDICAL CARE IN THE VETERAN'S ADMINISTRATION

In this section, we consider the rules that determine whether severely ill simulated cases of ILI become known to the VAMHCS system. Eligibility for services within the Veteran's Administration Health System is determined by whether or not the patient is a veteran. This has some obvious implications. For example, there are many fewer patients in the age-group, 0-19 years, in the care of the VAMHCS than would be expected based upon figures for the general US hospital population (See Figure 1). Patients in the age group, 0-19, years constitute less than 0.2% of the entire VAMHCS population whereas the same age group is 18% of the general hospital population. This leads to our first rule.

Rule 1.

We shall assume that simulated cases in patients less than 20 years old will NOT come to the

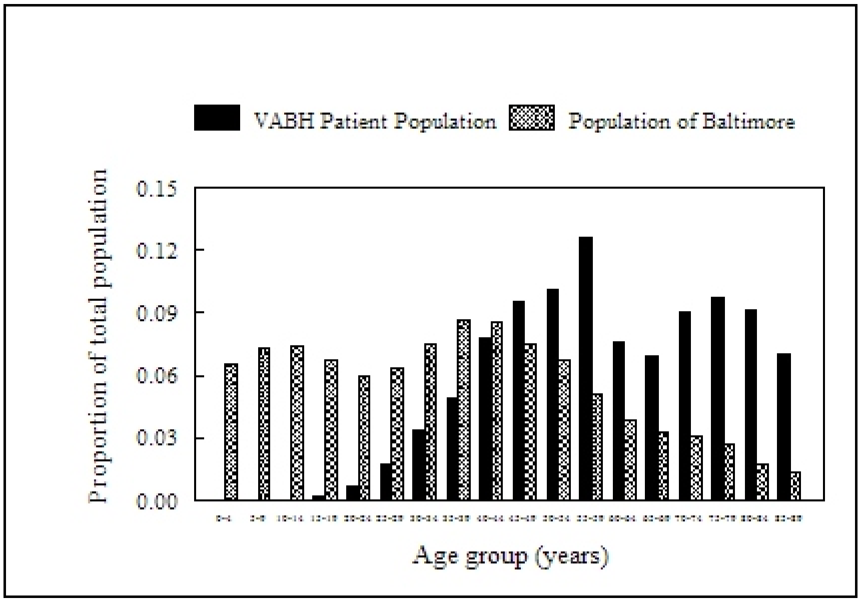

Figure C2. Data for VABH from Shawn Loftus (pers. Comm.); data for the population of Baltimore from the US Census, 2000.

attention of the VAMHCS.

THE FURTHER EFFECTS OF AGE, AND THE EFFECTS OF GENDER

The age-class distribution of patients known to the VAMHCS is not a simple linear function of the age-class distribution of the population of Baltimore (Figure C2). Older patients are over represented in the VAMHCS medical records.

Older patients are also over represented in national hospital patient populations (Elixhauser, Yu, Steiner, Bierman , 2000) , but, as we have already seen, the age-class distributions of the patient populations of the VAMHCS and the national hospital system are different.

Increasing age is a risk factor for hospitalization. Nationally, increasing age is a carrier variable (surrogate) for a host of age related factors that affect morbidity. However, within the VAMHCS, age also determines eligibility for care because the risk of being a veteran changes with age. This is one reason why the VAMHCS patient population is different from the national hospital population. The Department of Veteran's Affairs website publishes statistics for the proportion of the population that is eligible for care under Veteran's Administration. For example, in Baltimore County, 0.19 of males 18-64 years old are eligible for care and so are 0.71 males older than 65. (http://www.va.gov/vetdata/Census2000/index.htm). The corresponding figures for females are 0.013 females 18-64 years old, and 0.016 females older than 65.

Rule 2. Simulated cases in males over 20 years of age will come to the attention of the VAMHCS system with probability 0.19 if younger than 64 years and with probability 0.71 if 65 years old or older.

Rule 3. Simulated cases in females over 20 years of age will come to the attention of the VAMHCS with probability 0.013 if younger than 64 years and with probability 0.016 if 65 years

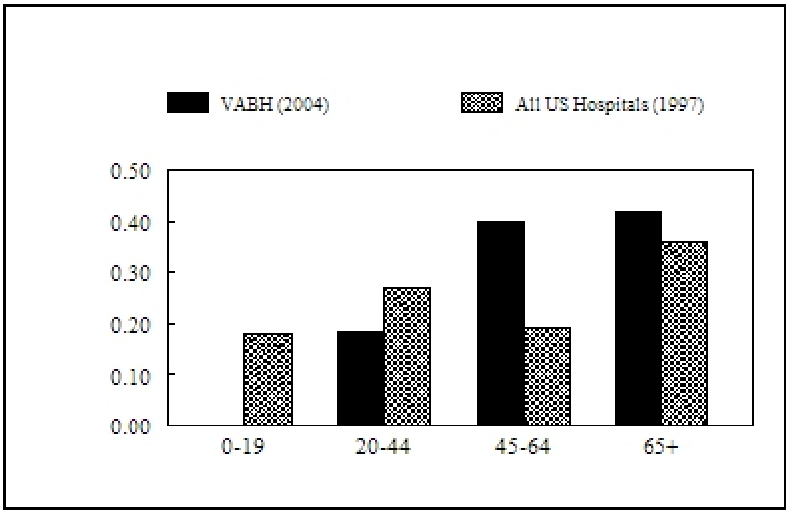

Figure C3. Age class distribution of patients cared for by the VABH system compared with the age-class distribution for the patients cared for by US hospitals in general. (Data for VABH from refer to 2004 and are from Shawn Loftus (*pers. comm.*); data for US hospital patient population refer to 1997 and are from Elixhauser, Yu, Steiner, Bierman (2000)

old or older.

DECISION TREE FOR DETERMINING THE PROBABILITY POR) THAT A CASE WILL BE RECORDED BY THE VAMHCS.

The conditional probability of a severely ill simulated ILI case being recorded by the VAMHCS can be estimated using the algorithm illustrated in Figure C4.

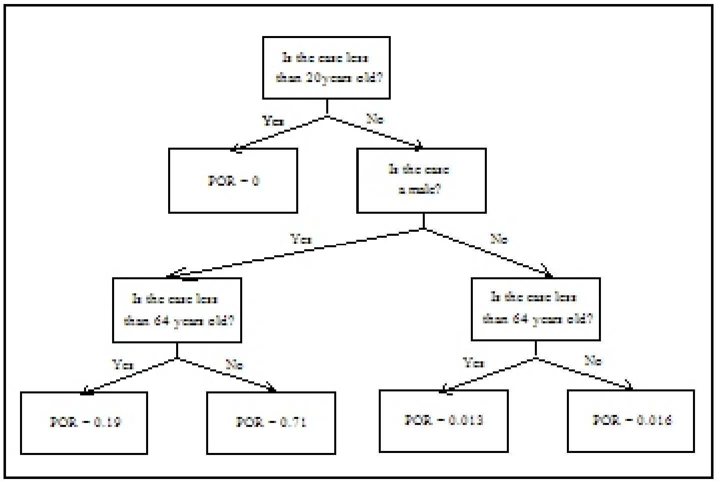

Figure C4 The conditional probability that a case will be recorded by the VABH system, See text for details.

THE RELEVANT FILES

Case_Generator5.m requires two data files*: Sorted_Results.xls* (Appendix B) and *Prob_of_Discovery.xls.*

Prob_of_Discovery.xls: Lists the relative probability of hospitalization, the relative probability of visiting an ER, and relative probability of visiting a clinic conditional on being a case of a specified age as reported by Menec *et al.*, (2003) (see above).

0.00214	0.00389	0.0518
0.00034	0.00042	0.0227
0.00537	0.0019	0.0376

These relative probabilities are the basis for the calculation of the absolute probabilities used by *Case_generator5.m* (se above)

Case_Generator5.m generates three results files: *Discovered_Cases.xls*, *VA_Recorded_Cases.xls*, and *Dataset.xls.*

Discovered_Cases.xls: This file contains all Discovered Cases grouped by five-year age-class, week number and zip code (columns 1-20: cases by age-group, column 21: zip code; column 22: total number of infectious people, column 23: week number). A few rows of columns 15-23 are printed below as an example.

1	0	1	0	0	21237	443.0513	14
11	4	3	0	0	21239	1876.738	14
16	6	9	4	0	21201	3416.195	15
17	10	7	2	0	21202	3339.92	15

VA_Recorded_Cases.xls: This file contains all Recorded Cases in VAMHCS medical record grouped by five-year age-class, week number and zip code (columns 1-20: cases by age-group, column 21:zip code; column 22: total number of infectious people , column 23: week number). The same few rows of columns 15- 23 are printed below so that *Discovered_Cases.xls* can be compared with *VA_Recorded_Cases.xls*.

1	0	0	0	0	21237	443.0513	14
7	1	1	0	0	21239	1876.738	14
7	2	5	3	0	21201	3416.195	15
4	4	1	2	0	21202	3339.92	15

Datasets.xls: Is the principal results file. This file consists of three columns: column 1is the number of new cases that become known to the VAMHCS; column 2 is the week number; column 3 is the zip code. A segment of the file is printed below.

2	3	21212
2	3	21222
2	4	21201
2	4	21208
2	4	21212
1	4	21222
2	4	21223
1	4	21224
1	4	21227
1	5	21201
1	5	21205
2	5	21212

Appendix D. The Date Generator

*Date_Generator5**a**.m* requires one data file: *Dataset.xls* (Appendix C)

*Date_Generator5**a**.m* generates one results file is called Jills_Data.xls.

Jills_Data.xls. consists of three columns (column 1 is the date in Excel date number format, column 2 is the ILI count, column 3 is the zip code). A segment of the file is printed below.

37641.00	14	21228
37641.00	21	21230
37641.00	10	21231
37641.00	4	21237
37641.00	10	21239

To convert the Excel serial number date format to something more familiar, simply open the file in Excel, highlight column 1, and using the format cell command click on “date” in the pop-up window. The result is as follows:

1/20/2003	14	21228
1/20/2003	21	21230
1/20/2003	10	21231
1/20/2003	4	21237
1/20/2003	10	21239

REFERENCES

Anderson, R.M. and May, R.M. (1991) *Infectious Diseases of Humans: Dynamics and Control* Oxford University Press, Oxford.

Bell, M.L. & Davis, D.L. (2001) reassessment of the lethal London Fog of 1952. Environmental Health Perspectives 109 (Suppl 3): 389-394.

Dobson, A. (2003) Metalife! Science 301: 1488-1490.

Donnelly, CA, Ferguson, NM, Ghani, AC, Woolhouse, MEJ, Watt CJ, Anderson RM (1997). The epidemiology of BSE in cattle herds in Great Britain. I. Epidemiological processes, demography of cattle and approaches to control by culling. Philosophical Transactions of the Royal Society of London, Series B 352: 781-804

Edmunds, WJ, O'Callaghan CJ, Noakes DJ. (1997) Who mixes with whom? A method to determine the contact pattern o adults that may lead to the spread of airborne infections. Proceedings of the Royal Society of London B 264: 949-957.

Elixhauser A, Yu K, Steiner C, Bierman AS. (2000) Hospitalization in the United States, 1997. Rockville (MD):Agency for Healthcare Research and Quality; HCUP Fact Book No. 1; AHRQ Publication No. 00-0031.ISBN 1-5876

Irwin DE, Weatherby LB, Huang W-Y, Rosenberg DM, Cook SF & Walker AM (2001) Impact of patient characteristics on the risk of influenza/ILI-related complications. BMC Health Services Research 1:8.

Ferguson, NM, Donnelly, CS, & Anderson, RM. (2001a) The foot-and-mouth epidemic in Great Britain: pattern of spread and impact of interventions 292 115-1160.

Ferguson, NM, Donnelly, CS, & Anderson, RM. (2001b) Transmission intensity and impact of control policies on the foot and mouth epidemic in Great Britain Nature 413 542-548.

Freckleton, R. P. and Watkinson A. R. (2002) Large scale spatial dynamics of plants: metapopulations, regional ensembles and patchy populations. Journal of Ecology 90: 419-434.

Gilpin, M. and Hanski, I. (1991) *Metapopulation dynamics: empirical and theoretical investigations*. Academic Press.

Grenfell, B. and Harwood, J. (1997) (Meta)population dynamics of infectious diseases. Trends in Ecology and Evolution 12: 395-399.

Guyer, B., Hoyert, D.L., Martin, J.A., Ventura, S.J., MacDorman, M..F, & Strobino, D.M. (1999)

Annual Summary of Vital Statistics - 1998. Pediatrics. 104 No 6 December 1999: 1229-1246.(Table 3).

Hak E, Wei F, Nordin J, Mullooly J, Poblete S, Nichiol KL (2004) development and validation of a clinical prediction rule for hospitalization due to pneumonia or influenza during influenza epidemics among community-dwelling elderly persons. Journal of Infectious Disease 189: 450-458.

Hanski, I. (1999) *Metapopulation Ecology*. Oxford University Press, Oxford.

Keeling, MJ, Woolhouse, MEJ, Shaw, DJ, Matthews, L, Chase-Topping, M, Haydon, DT, Cornell, SJ, Kappey, J, Wilesmith, J & Grenfell, BT. (2001) Dynamics of the 2001 UK Foot and Mouth Epidemic: stochastic dispersal in a heterogeneous landscape Science 294 813-817.

Keeling, M. J. and Gilligan, C. A. (2000) Metapopulation dynamics of bubonic plague. Nature 407: 903-906.

Keeling, MJ, Woolhouse, MEJ, May, RM, Davies, G & Grenfell, BT. (2003) Modelling vaccination strategies against foot-and-mouth disease. Nature 421 136-142.

Keeling, MJ, Bjornstad, OJ, Grenfell, BT. (2004) Metapopulation Dynamics of Infectious Diseases In Ecology, Genetics and Evolution of Metapopulations. Elsevier Academic Press.

Keeling. MJ. (1997) Modelling the persistence of measles. Trends in Microbiology 5: 513-518

Mamalund, S-E. (2001) "The Spanish Influenza among Norwegian Ethnic Minorities" Center for Demography and Ecology Working Paper No. 2001-11, University of Wisconsin.

Matthews, L., Haydon, D. T., Shaw, D. J., Chase-Topping, M. E., Keeling, M. J. and Woolhouse, M. E. J. (2003) Neighborhood control policies and the spread of infectious disease. Proceedings of the Royal Society of London Series B. 270: 1659-1666.

McLean, A.R. & Anderson, R.M. (1988) Measles in developing countries. Part I. Epidemiological Parameters and Patterns. Epidemiology and Infection. 100: 111-133.

Menec VH, Black C, MacWilliam L, Aoki FY (2003) The impact of influenza associated respiratory illness on hospitalizations, physician visits and mortality. Canadian Journal of Public health 94: 59-63.

Morris, RS, Wilesmith, JW, Stern, MW, Sanson, RL, & Stevenson, MA. 2001 . Predictive spatial modelling of alternative control strategies for the foot-and-mouth disease epidemic in Great Britain Vet. Record 149 137-144.

Reis ,B.Y., Pagano, M., Mandl, K.D. (2003) Using temporal context to improve biosurveillance. Proceedings of the National Academy of Sciences 100: 1961-1965.

Smith, G. & Scott, M. E. (1994) Model behavior and the basic reproduction ratio In: Scott M. E. and Smith G. (eds) *Parasitic and Infectious Disease: Epidemiology and Ecology*. Academic Press, San Diego 21-28.

Sprenger MJ, Mulder PG, Beyer WE, Van Strik R, Masurel N (1993) Impact of influenza on mortality in relation to age and underlying disease. International Journal of Epidemiology 22: 334-340.

Thomas, C. D. and Kunin W. E. (1999) The spatial structure of populations. Journal of Animal Ecology 68: 647-657.

Thompson WW, Shay DK, Weintraub E, Brammer L, Bridges CB, Cox N Fukuda K (2004). Influenza-associated hospitalizations in the United States. Journal of the American Medical Association. 292: 1333-1340

Thompson WW, Shay DK, Weintraub E, Brammer L, Cox N, Anderson LJ, Fukuda K (2003a). Mortality associated with influenza and respiratory syncytial virus in the United States. Journal of the American Medical Association. 289: 179-186.

Woolhouse, MEJ & Donaldson, A. (2001) Managing foot-and-mouth. The science of controlling disease outbreaks Nature 515-516
